# Implications of *CLSPN* Variants in Cellular Function and Susceptibility to Cancer

**DOI:** 10.3390/cancers12092396

**Published:** 2020-08-24

**Authors:** Diana Azenha, Santiago Hernandez-Perez, Yuse Martin, Marta S. Viegas, Alexandra Martins, Maria C. Lopes, Eric W. -F. Lam, Raimundo Freire, Teresa C. Martins

**Affiliations:** 1 Faculdade de Farmácia da Universidade de Coimbra, 3000-548 Coimbra, Portugal; diana.azenha@vetdiagnos.pt (D.A.); celeste@ff.uc.pt (M.C.L.); 2 New preventive and therapeutic strategies, Centro de Neurociências e Biologia Celular, Universidade de Coimbra, 3004-504 Coimbra, Portugal; marta.s.viegas@gmail.com; 3 Radiobiology, Department of Medical Physics, Instituto Português de Oncologia de Coimbra de Francisco Gentil, 3000-651 Coimbra, Portugal; 4 Unidad de Investigación, Hospital Universitario de Canarias, 38320 Tenerife, Spain; s.hernandez.perez@nki.nl (S.H.-P.); yusemartin@gmail.com (Y.M.); rfreire@ull.edu.es (R.F.); 5 Molecular Pathology Laboratory, Instituto Português de Oncologia de Coimbra de Francisco Gentil, 3000-651 Coimbra, Portugal; 6 Inserm U1245, UNIROUEN, Normandie Univ, Normandy Centre for Genomic and Personalized Medicine, 76183 Rouen, France; alexandra.martins@univ-rouen.fr; 7 Department of Surgery and Cancer, Imperial College London, Imperial Centre for Translational and Experimental Medicine (ICTEM), London W12 0NN, UK; eric.lam@imperial.ac.uk; 8 Instituto de Tecnologías Biomédicas, Universidad de La Laguna, 38200 Tenerife, Spain; 9 Universidad Fernando Pessoa Canarias, 35450 Las Palmas de Gran Canaria, Spain

**Keywords:** Claspin, genetic changes, cancer, breast cancer, glioma

## Abstract

Claspin is a multifunctional protein that participates in physiological processes essential for cell homeostasis that are often defective in cancer, namely due to genetic changes. It is conceivable that Claspin gene (*CLSPN*) alterations may contribute to cancer development. Therefore, *CLSPN* germline alterations were characterized in sporadic and familial breast cancer and glioma samples, as well as in six cancer cell lines. Their association to cancer susceptibility and functional impact were investigated. Eight variants were identified (c.-68C>T, c.17G>A, c.1574A>G, c.2230T>C, c.2028+16G>A, c.3595-3597del, and c.3839C>T). *CLSPN* c.1574A>G (p.Asn525Ser) was significantly associated with breast cancer and was shown to cause partial exon skipping and decreased Claspin expression and Chk1 activation in a minigene splicing assay and in signalling experiments, respectively. *CLSPN* c.2028+16G>A was significantly associated with familial breast cancer and glioma, whereas c.2230T>C (p.Ser744Pro), was exclusively detected in breast cancer and glioma patients, but not in healthy controls. The remaining variants lacked a significant association with cancer. Nevertheless, the c.-68C>T promoter variant increased transcriptional activity in a luciferase assay. In conclusion, some of the *CLSPN* variants identified in the present study appear to modulate Claspin’s function by altering *CLSPN* transcription and RNA processing, as well as Chk1 activation.

## 1. Introduction

Cancer cells derive from the transformation of healthy cells after progressive and cumulative acquisition of genetic and epigenetic alterations that affect essential cellular functions [1,2]. Genomic instability lies at the basis of malignant transformation and occurs as a consequence of deficiencies in genome protective mechanisms, such as cell cycle checkpoints, DNA replication control or DNA repair [1,2,3].

Claspin integrates the functions of a group of crucial proteins with the maintenance of genome stability [4], by being an important player in key cellular events such as checkpoint activation after DNA damage, DNA replication and replication stress responses, DNA repair and apoptosis [5,6,7]. Checkpoint activation after DNA damage provides cells time to repair the damage before cell cycle progression. If this is not possible, checkpoints are deactivated and the cells induced to die, namely by apoptosis. One of the major mediators of checkpoint activation is Chk1. Claspin is required for Chk1 activation following DNA damage in both Xenopus and mammalian cells, mediating Chk1 phosphorylation by ATR [8,9,10,11]. Although research regarding the role of Claspin in DNA repair is still scarce, Claspin has already been implicated in several DNA repair mechanisms [12,13,14,15,16,17,18]. Moreover, Claspin degradation is required for the termination of Chk1-dependent checkpoint activation and for apoptosis induction when DNA damage becomes irreparable [19,20].

Claspin also has a role in DNA replication. It has a ring-shaped DNA binding structure that shows high affinity for replication associated structures [21,22] and interacts with numerous replication proteins [21,22,23,24,25], being an integral component of the replisome [5,21,22,24,26]. Claspin is required for ensuring normal rates of replication [27,28] and functions together with Cdc7 to regulate origin firing [29,30]. Replication fidelity is of paramount importance for the maintenance of genome integrity. Claspin together with Timeless and Tipin form a “replication fork protection complex” that participates in several functions involved in the protection of DNA replication [22,31,32,33,34].

Given the crucial functions of Claspin in genomic protection and cell homeostasis, one can predict a role for Claspin in cancer. In fact, there is a considerable body of evidence linking Claspin deregulation with cancer [6]. For instance, a recent study demonstrated that overexpression of Claspin and Timeless promoted survival of cancer cells by enabling adaptation to oncogene-induced replication stress [35]. In addition, Claspin gene (*CLSPN*) mutations that affect checkpoint regulation were identified in cancer patients suggesting that they may contribute to cancer development [36,37,38,39]. The *CLSPN* I783S missense mutation, identified in an ovarian tumour-derived cell line, has been linked to defective Chk1 phosphorylation following UV exposure [36].

In this study, we characterized the spectrum of *CLSPN* genetic alterations present in breast cancer and glioma samples and cell lines, and assessed their association with cancer, as well as their possible functional consequences. We found several *CLSPN* variants associated with breast cancer and glioma. Functional studies showed that *CLSPN* variants may impact Claspin expression and function. Our data suggest that *CLSPN* genetic alterations may affect *CLSPN* transcription and RNA processing and, as a consequence, impact Claspin function. However, the effects of these changes in cancer development remain to be assessed.

## 2. Results

### 2.1. CLSPN Alterations in Breast Cancer and Glioma Patients

We began by characterizing the spectrum of *CLSPN* germline alterations occurring in familial breast cancer (*n* = 147), sporadic breast cancer (*n* = 66) and in glioma (*n* = 53) patients. A control group, consisting of healthy women with no oncologic history, was also included in the study (*n* = 79). The *CLSPN* germline variants detected in breast cancer and glioma patients are presented in Table 1.

We next analysed the genotypic and allelic frequencies of the *CLSPN* variants, as well as their possible association with cancer. The allelic frequency of c.1574A>G (p.Asn525Ser) was of 13.6% in the familial breast cancer group, 8.3% in the sporadic breast cancer group and 14.2% in the glioma group (Table 2). c.1574A>G allele frequency in our control group was of 22.8% whereas the non-Finnish European allele frequency was 12.9% (gnomAD v2.1.1. for rs7537203, Cambridge, MA, USA). We also found that *CLSPN* c.1574A>G (p.Asn525Ser) was significantly associated with both familial and sporadic forms of breast cancer with an overrepresentation of the A allele (Table 2). However, no association was found in glioma.

*CLSPN* c.2028+16G>A was significantly associated with familial breast cancer, with a higher frequency of the G allele among familial breast cancer cases when compared to controls. Statistically significant differences were found when comparing c.2028+16G>A allelic frequencies between controls and the glioma group (*p* < 0.05). The data revealed an overrepresentation of c.2028+16A allele among glioma patients (Table 2). However, we did not detect any differences when comparing genotypic frequencies, although a trend toward significance was observed (*p* = 0.0615). The c.2028+16G>A allele frequency in our control group was of 74.1%, whereas non-Finnish European allele frequency was 87.1% (gnomAD v2.1.1. for rs535638).

As for the genotypic and allele frequencies of *CLSPN* c.-68C>T, c.17G>A, c.2230T>C, c.3595-3597delGAA and c.3839C>T variants, no major differences were detected between controls and cancer patients (Tables S1 and S2).

In our study, the *CLSPN* c.2230T>C variant was only detected in cancer patients (Table S1). c.2230T>C allele frequency was of 0.7% in the familial breast cancer group, 0.8% in the sporadic breast cancer group and 0.9% in the glioma group and no homozygous individuals were identified (Table S1). c.2230T>C allele frequency in Exomes and Genomes projects was 0.004651% (gnomAD v2.1.1. for rs753369867). These data suggest that c.2230T>C is exclusively found in cancer patients and thus associated with the disease.

### 2.2. CLSPN Alterations in Cell Lines

In order to try to isolate more *CLSPN* variants associated with cancer, we also decided to characterize a set of cell lines commonly used as in vitro cancer models (Table 3). *CLSPN* c.2028+16G>A variant was never detected in the homozygous wild type (WT) form (GG) and its heterozygous form (GA) appears to segregate with c.1574A>G heterozygous form (AG) (Table 3).

### 2.3. CLSPN c.1574A>G Variant

As we found a significant association of the c.1574A>G variant with breast cancer, we decided next to study its functional impact. For that, we first used predictive bioinformatics tools. Polyphen-2 (Cambridge, MA, USA) predicted *CLSPN* c.1574A>G (p.Asn525Ser) variant to be probably damaging (score of 0.994). We then calculated total ESR score changes (ΔtESRseq scores) to predict the effect of single exonic variations on potential exonic splicing regulatory elements, such as exonic splicing enhancers (ESEs) and silencers (ESS) [40,41]. The total ESR score change for c.1574A>G was −1.8347, suggesting that this variant may induce exon skipping (ΔESRseq score < −0.66 being considered damaging [41]). Finally, we used Alamut Visual (Rouen, France) interface to predict the effect of *CLSPN* variants on canonical 3′ and 5′ splice sites, that is, *CLSPN* natural splice sites. Alamut analysis indicated that exon 8 *CLSPN* c.1574A>G variant does not cause a direct alteration of the strength of the reference 5′ splice site. Skipping of exon 8 (r.1005_1579del) produces a frameshift leading to the introduction of a premature stop codon. Such aberrant transcript is predicted to either be degraded by the nonsense-mediated RNA degradation pathway and/or to generate a truncated Claspin protein (p.Ser336Thrfs*13).

**Figure 1:**
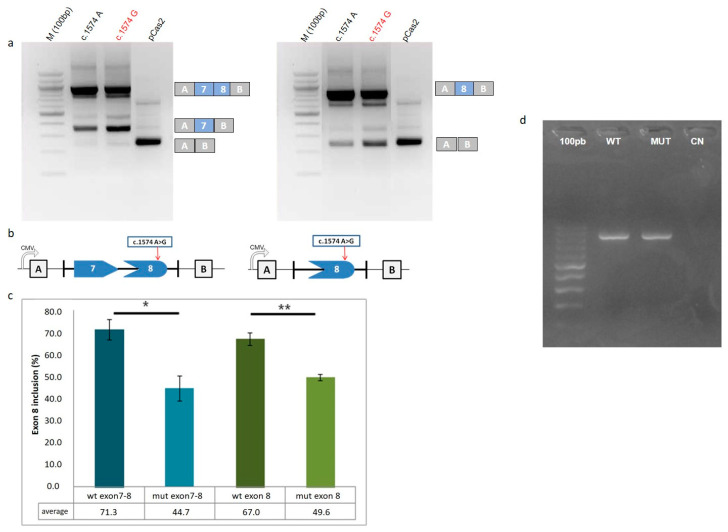
*CLSPN* c.1574A>G variant increases the probability of exon 8 skipping in vitro. pCAS2 empty vector (pCAS2) or pCAS2 constructs carrying exonic fragments with *CLSPN* c.1574A>G variant were transfected in HeLa cells. Total RNA was extracted 24 h after transfection and RT-PCR was performed. RT-PCR products were separated on a 2% agarose gel stained with ethidium bromide and visualized under UV light. (**a**) *CLSPN* c.1574A>G leads to exon 8 skipping, both in single (exon 8) and double exon (exon 7 and 8) minigene constructs. The identities of the RT-PCR products are indicated on the right. (**b**) Schematic representation of minigene splicing reporter vector. Exon 7 + 8 or exon 8 were inserted into pCAS2 vector. The arrow indicates the position of the variant. (**c**) Quantity One analysis software was used to quantify RT-PCR bands and assess exon 8 inclusion, both in the WT and mutant forms of the double and single minigene structures. Mean+SD of exon 8 inclusion were calculated for each minigene. The graphic shows the average percentages of exon 8 inclusion obtained in three independent experiments. Statistical analysis of the results was performed using Student’s *t* Test. (**d**) *CLSPN* c.1574A>G is not associated with the production of aberrantly spliced transcripts in HeLa cells. Electrophoretic profile obtained after RT-PCR analysis of RNA from HEK293 (WT) and HeLa (MUT) (heterozygous for *CLSPN* c.1574A>G) cells. Only the expected RT-PCR amplicon (822 bp) was obtained for both the WT and mutant samples. A 100 bp DNA ladder was used (100 bp); CN, negative control. The image displayed represents one of the three experiments performed. * *p* < 0.05; ** *p* < 0.001. Detailed information about western blot can be found at Figure S1.

Based on these findings, we then experimentally assessed the impact of *CLSPN* c.1574A>G on splicing using minigene assays. We showed that the double-exon WT *CLSPN* minigene (pCAS2.*CLSPN* exons 7-8 c.1574 A) produced a higher level of transcripts containing exon 8 than the mutant form c.1574 G (71.3% versus 44.7%, respectively) (Figure 1), which displayed an increase in exon skipping. Similar results were obtained for the c.1574A>G single exon pCAS2 reporter construct. In this context, we observed that the WT form had 67.0% of exon 8 inclusion, whereas the mutant form only had 49.6% due to an increase in exon skipping (Figure 1). Altogether, these data indicate that *CLSPN* c.1574A>G increases skipping of exon 8 and, consequently, may affect Claspin’s structure and function.

We next investigated whether *CLSPN* c.1574A>G variant is associated with the production of distinct *CLSPN* transcripts, as a result of aberrant splicing. Given that HeLa cells are heterozygous for *CLSPN* c.1574A>G and HEK293 cells are WT (Table 3), their RNAs were used as RT-PCR templates. RT-PCR was performed using exonic primers flanking the variants and the size and sequence of the resulting amplicons (WT and mutant) were compared. As shown in Figure 1d, all the amplicons obtained were of the expected size. Sequencing revealed that the WT and variant containing amplicons were identical. Additional bands that could suggest aberrant splicing were not detected. Therefore, our data suggest that either c.1574A>G is not causative of aberrant splicing or that eventually generated aberrant transcripts are scarce or degraded and thus very difficult to detect.

*Cis*-regulatory elements can affect gene expression in an allele specific manner. Allelic imbalance occurs whenever one allele of a heterozygous variant is more expressed than the other, which suggests the action of *cis*-regulatory elements. *CLSPN* c.1574A>G was significantly associated with cancer in our study. To investigate if this could be due to *cis*-acting factors, c.1574A>G allelic imbalance (AI) was assessed. For that, a SNaPshot assay was performed. SNaPshot assays measure the ratio between the amounts of labelled nucleotides incorporated in primer extension reactions for the two alleles in mRNA (cDNA). This ratio was then compared to the ratio measured in gDNA, where the two alleles are present in an equimolar ratio.

**Figure 2:**
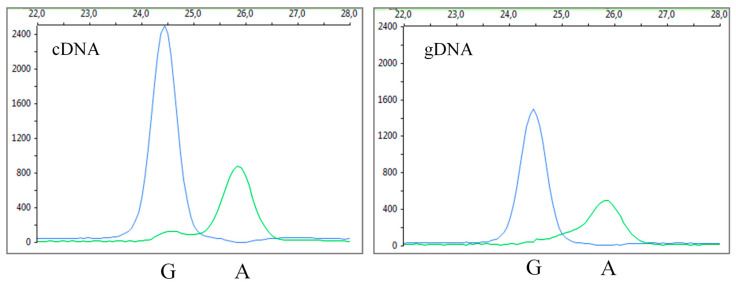
There is no evidence of allelic imbalance associated with *CLSPN* c.1574A>G in HeLa cells. Electropherograms depicting fluorescence peaks obtained from HeLa cDNA (left panel) and gDNA (right panel) primer extension analysis. Allelic variants are labelled at the bottom of their corresponding peak. X axis: size in base pairs; Y axis: fluorescence intensity. The average of the normalized ratio cDNA (WT/Mut)/ gDNA (WT/Mut) obtained was 0.9 ± 0.1, which is indicative of no AI.

In this study, we used the HeLa cells since these cells are heterozygous for the *CLSPN* c.1574A>G variant. Taking into account only the cDNA ratio (WT/Mut), significant AI was detected (0.43). However, after normalization to gDNA (WT/Mut) ratio, *CLSPN* c.1574A>G allelic ratios did not deviate significantly from the unit (Figure 2). The average of the normalized ratio cDNA (WT/Mut)/gDNA (WT/Mut) obtained was 0.9 ± 0.1. Therefore, it can be concluded that there is no evidence of AI for the *CLSPN* c.1574A>G in HeLa cells.

**Figure 3:**
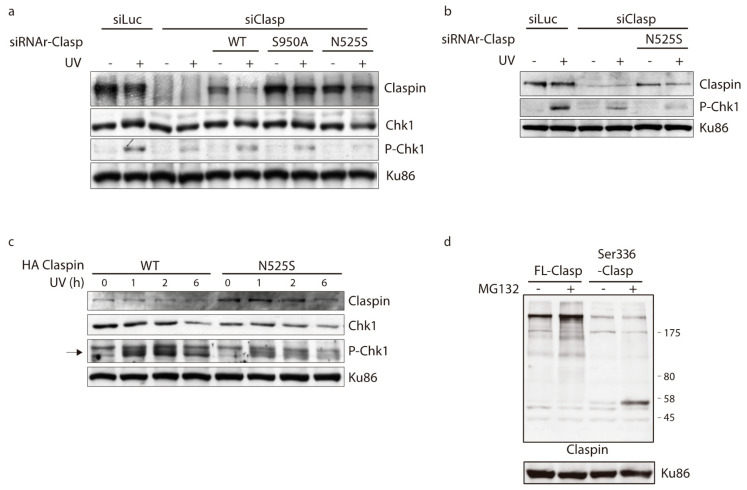
Claspin c.1574A>G mutants, p.N525S and p.Ser336Thrfs*13, impact Claspin expression and function. (**a**) and (**b**) U2 OS cell lines stably expressing HA tagged WT, S950A or N525S versions of Claspin, also containing silent mutations to the small interfering RNA, were transfected with siRNA against Claspin to deplete endogenous Claspin. The cells were then treated with UV (40 J/m^2^) and collected at 1-h post irradiation to be analysed by Western blot against the indicated antibodies. (**c**) a similar experiment to (**a**) and (**b**) was performed with the stable cell lines expressing WT and N525S versions of Claspin and cells were collected at the indicated time points after UV treatment (40 J/m^2^); the arrow indicates the phosphorylated form of Chk1 at Serine 317. (**d**) Full length or Ser336 Flag expressing plasmids were transfected in HEK293T cells and 24 h after transfection were incubated when indicated with the MG132 proteasome inhibitor. Protein Ku86 was included as loading control in all blots. Detailed information about western blot can be found at Figure S2.

As bioinformatics data suggested a potential functional impact for the c.1574A>G variant and this variant would result in an amino acid (aa) change at position 525, from an Asparagine (Asn) to a Serine (Ser) (p.N525S), we decided to investigate whether c.1574A>G could affect Claspin function, namely with regard to Chk1 activation. To this end, we obtained stable cell lines expressing a version of HA-Claspin that is resistant to siRNA treatment, which included, or not, the N525S mutation. As Claspin is required for the activation of Chk1 after DNA damage, we tested if the N525S mutation could affect Chk1 phosphorylation. We have also tried to obtain a Claspin S744P expressing cell line, but without success. Nevertheless, we obtained a Claspin containing the S950A mutation, which we had also detected in cancer samples in early stages of the herein presented work. However, later on, this variant turned out not to be significantly associated with cancer. As shown in Figure 3a,b, the N525S substitution decreased Chk1 phosphorylation in Ser317 with regard to cells expressing WT Claspin or the S950A mutation. Also as expected, deletion of endogenous Claspin decreased Chk1 activation after DNA damage. To investigate if the N525S mutation had any effect in Chk1 phosphorylation in longer time points after DNA damage, we carried out a similar experiment at different times post irradiation (Figure 3c). Indeed, Chk1 phosphorylation was decreased at later time points after DNA damage in Claspin N525S expressing cells. Moreover, the effect of N525S mutation in Chk1 activation could not be attributed to higher Claspin instability as Claspin N525S levels did not change in the times post irradiation studied.

We also investigated the effect of the p.Ser336Thrfs*13 alteration (corresponding to *CLSPN* transcripts lacking exon 8) on Claspin function in a similar set up. However, we were not able to detect a tagged version containing the expected fragment. As Claspin is a protein that is heavily regulated by ubiquitination and proteasome degradation, we therefore decided to study the expression of the fragment in conditions of proteasome inhibition by adding MG132 to the media. Interestingly, we were able to detect the expected fragment with an antibody raised against a N-terminal segment of Claspin (Figure 3b). This suggests that the p.Ser336Thrfs*13 substitution leads to the production of a truncated protein that is very unstable, and may, therefore, behave as a null mutation.

Taken together, our data suggest that the c.1574A>G variant impacts Claspin expression and Chk1 activation. The p.N525S form is associated with defective Chk1 activation, whereas the p.Ser336 Thrfs*13 form leads to the production of a very unstable truncated Claspin protein, and therefore acts as null mutation.

### 2.4. Evaluation of the Impact of CLSPN Promoter c.-68C>T Mutation

**Figure 4:**
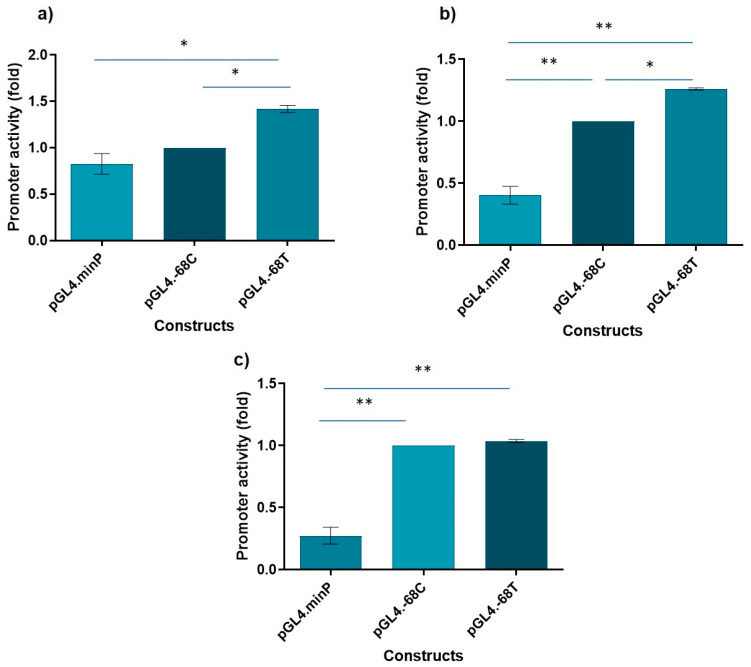
The c.-68C>T *CLSPN* promoter variant increases transcriptional activity in HEK293 and HeLa cell lines. After cloning a 150 bp fragment of the *CLSPN* promoter containing the variant into a minimal promoter (minP) vector, luciferase assays were performed to assess the transcriptional activity associated with each allele. A higher transcriptional activity was observed for the T allele in HEK293 (**a**) and HeLa (**b**) cells. No significant differences were observed in U2 OS cells (**c**). Experiments were performed three times independently and in triplicate. All data were combined for statistical analysis. * *p* < 0.001; ** *p* < 0.0001.

We previously identified a promoter variant, c.-68C>T, in both healthy and cancer patients [39]. The c.-68C>T allele frequency in the control group was 0.6%, whereas in familial and sporadic breast cancer and glioma groups the allele frequencies were of 1.4%, 0.8% and 2.8%, respectively. The allele frequency in the non-Finnish European population is 0.2984% (gnomAD v2.1.1. for rs372789882). Although no statistically significant association was found with cancer, promoter mutations can modulate gene transcription, being thus important to assess their impact. Therefore, we investigated whether the c.-68C>T variant could alter the activity of the *CLSPN* promoter. For that, we performed luciferase assays using HEK293, HeLa and U2 OS cell lines. We observed that the mutant allele (c.-68T) had a significantly higher transcriptional activity than the WT allele (c.-68C) in both HEK293 (41.8%) and Hela (26.1%) cell lines, suggesting that the c.-68C>T variant may improve *CLSPN* promoter activity (Figure 4a,b). Regarding the U2 OS cell line, no differences were found (Figure 4c).

**Figure 5:**
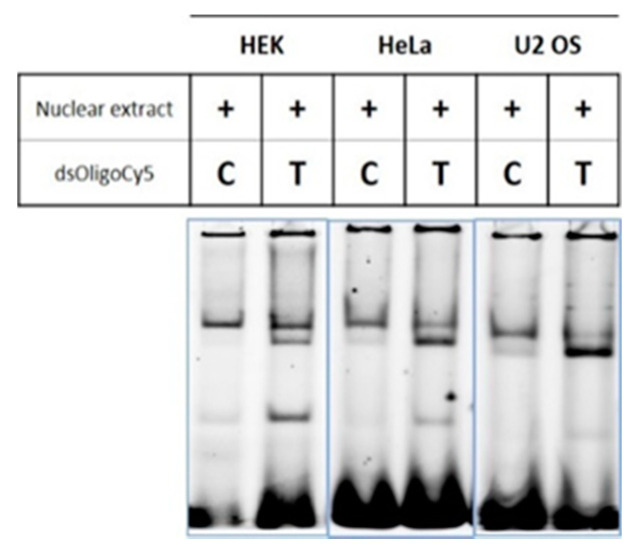
The Electrophoretic Mobility Shift Assay (EMSA) profiles of the C and T alleles of c.-68C>T variant are different. EMSA was performed using labelled oligonucleotides (dsOligoCy5) containing the C or T allele corresponding to *CLSPN* c.-68C>T. The oligonucleotides were incubated with nuclear extracts from HEK293, HeLa and U2 OS cells. Each band corresponds to a distinct protein-DNA complex formed during incubation of nuclear extracts with the labelled oligonucleotides. Unbound/free oligonucleotides are detected at the bottom of the gel. The assays were repeated at least three times for each cell line and a representative experiment is shown. Detailed information can be found at Figure S3.

As the activity of promoters can be modulated by the binding of transcription factors, the increased transcriptional activity observed for c.-68T allele prompted us to further investigate if this allele could alter the binding of transcription factors to the *CLSPN* promoter. For that, we performed an Electrophoretic Mobility Shift Assay (EMSA) using nuclear extracts from HEK293, HeLa and U2 OS cell lines. We found that, while the C allele profile presented a single band, the one of the T allele presented three distinct bands (Figure 5), which suggests binding of different factors to each of the alleles.

**Figure 6:**
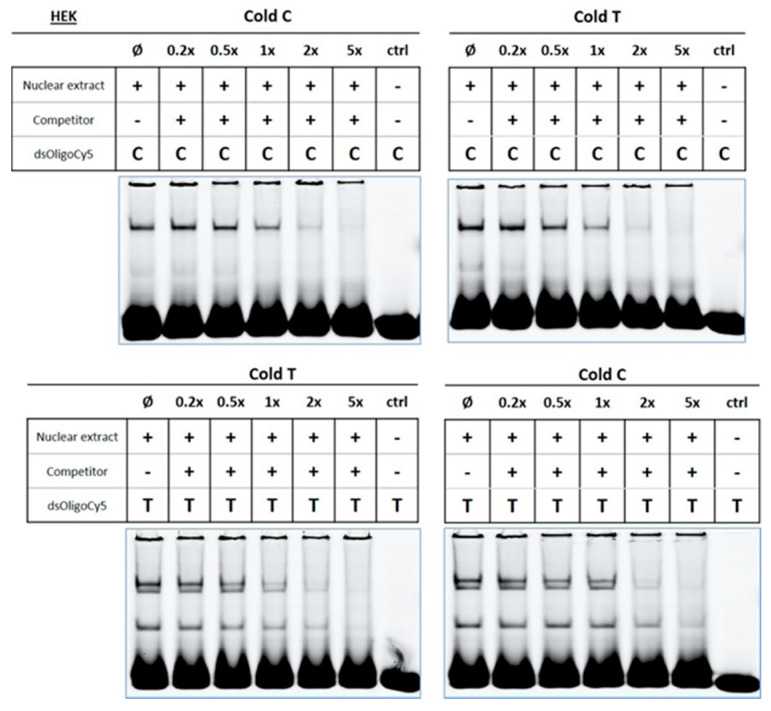
*CLSPN* c.-68C>T variant might alter transcription factor binding stoichiometry. Competition assays were performed with labelled oligonucleotides (dsOligoCy5) containing the C or T allele corresponding to *CLSPN* c.-68C>T. Where indicated (+) cold competitor probes containing either C or T were added to the reaction in a 0.2-, 0.5-, 1-, 2- or 5-fold excess, and their effect compared to the null reaction (Ø lane—no competitor added). The last lane of each assay corresponds to a reaction in which only the labelled oligonucleotide was added (ctrl). A representative assay for HEK293 cells is shown. The assay was also performed for HeLa and U2 OS cell lines (Figures S4 and S5). The assays were repeated at least three times for each cell line. Detailed information can be found at Figure S6.

We next performed competition assays using unlabelled oligos (cold probes). Competition assays are useful for assessing the specificity of binding. We found that competition assays with cold probes for both oligos (tested as cold C, with labelled C; and cold T, with labelled T) decreased the band intensity of the profile, an observation that is indicative of the specificity of the reaction (Figure 6). Of note, when competition assays were performed in which cold C probe was tested with labelled T, or cold T with labelled C, we also observed a decrease in the intensity of the band profile, a finding that suggests that the transcription factor(s) that binds to the C and T allele may be the same. Results obtained were similar for the three cell lines used. Therefore, only a representative experiment is shown. Together our data suggest that, although c.-68C>T variant may alter the binding of transcription factors, it may do so by altering the binding stoichiometry of the transcription factors rather than altering the type of transcription factor.

**Figure 7:**
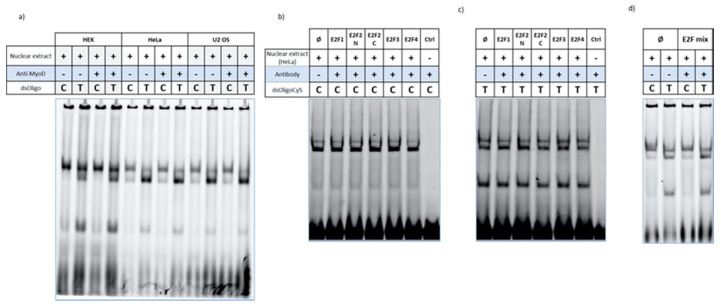
MyoD and E2F proteins do not bind to c.-68C>T site. No supershift was detected using anti-MyoD or anti-E2F antibodies. Where indicated (+) MyoD (**a**) or E2F antibodies (**b**–**d**) were added to the reaction and the effect compared with the reaction without the antibodies (lane Ø or -) for both alleles (C and T). The assays were repeated at least three times for HEK293, HeLa and U2 OS cell lines. A representative experiment is shown. E2F antibodies were tested either individually (**b**,**c**) or pooled (**d**). The last lane of each assay corresponds to reactions with oligonucleotides only (ctrl). Detailed information can be found at Figure S7.

Next, we tried to identify the transcription factor(s) involved. We first used bioinformatics tools to screen the region surrounding the *CLSPN* promoter mutation for transcription factor binding sequences. Transcription factor binding to c.-68C>T mutation site was compared between WT and mutant alleles. Transcription factors exclusively detected in the rare allele were considered as putative candidates for EMSA analysis. Software analysis yielded two candidate transcription factors: MyoD and myogenin. We selected MyoD as a candidate transcription factor due to a significant support from literature linking MyoD to breast cancer [42,43,44]. The transcription factors of the E2F family were also tested because *CLSPN* promoter is a bona fide target of these proteins [45,46]. To assess if MyoD and E2F transcription factors were responsible for the *CLSPN* c.-68C>T profile obtained in EMSA, supershift reactions were performed. HEK293, HeLa and U2 OS nuclear extracts were incubated with C or T oligonucleotides and with the antibodies directed to the aforementioned proteins. Antibodies to E2F proteins (E2F1, E2F2 N, E2F2 C, E2F3 and E2F4) were used either individually or pooled. No supershift bands were detected for any the cell lines tested for either MyoD (Figure 7a), or E2F proteins (Figure 7b–d). These data do not support a role for either MyoD or E2F proteins in c.-68C>T associated impact on *CLSPN* transcriptional activity and suggest that other transcription factors may be involved.

### 2.5. Other CLSPN Variants: c.17G>A (p.Gly6Asp), c.2028+16G>A, c.2230T>C (p.Ser744Pro), c.3595-3597delGAA (p.Glu1199del) and c.3839C>T (p.Ser1280Leu)

We also investigated the functional impact of other *CLSPN* variants, namely c.17G>A (p.Gly6Asp), c.2028+16G>A, c.2230T>C (p.Ser744Pro), c.3595-3597delGAA (p.Glu1199del) and c.3839C>T (p.Ser1280Leu). Bioinformatics tools were used to predict the functional impact of these variants. With the exception of *CLSPN* c.17G>A (p.Gly6Asp), which did not present a significant association with cancer and was not predicted to have a functional impact, the impact of all other variants on splicing was studied using minigene assays. However, no consistent or statistically significant results were found. The c.2028+16G>A variant was associated with glioma but we were not able to find any functional impact for this variant. We have also observed that the c.2230T>C variant (p.Ser744Pro) was exclusively detected in cancer samples, a finding that suggested a role for this variant in cancer. Unfortunately, we failed to obtain a stable Ser744Pro clone to test the impact of this variant on Claspin expression and Chk1 phosphorylation.

## 3. Discussion

Claspin has an important role in the maintenance of cell homeostasis, participating in crucial functions, such as the DNA damage response and cell cycle checkpoint regulation, as well as DNA replication and repair [6]. Claspin is well conserved among mammalian species, which is in agreement with its essential role in several physiological mechanisms. Due to the role of Claspin in overall cell homeostasis, it is reasonable to hypothesize that *CLSPN* genetic variants that affect Claspin function may play a role in cancer development. Therefore, in this study, *CLSPN* variants were investigated regarding their functional impact and potential contribution towards cancer susceptibility. For that, DNA from breast cancer (familial and sporadic forms) and glioma patients, as well as DNA from blood samples of a group of healthy individuals, were screened for *CLSPN* genetic alterations. Eight germline variants were found namely c.-68C>T, c.17G>A (p.Gly6Asp), c.1574A>G (p.Asn525Ser), c.2028+16G>A, c.2230T>C (p.Ser744Pro), c.3595-3597delGAA (p.Glu1199del) and c.3839C>T (p.Ser1280Leu). We found that the c.1574A>G (p.Asn525Ser) variant was associated with sporadic and familial breast cancer, whereas c.2028+16G>A was linked to glioma. The c.2230T>C (p.Ser744Pro) variant was exclusively detected in cancer patients, a finding that suggests that this variant may have impact on cancer development. Analysis of the allelic frequency of c.1574A>G (p.Asn525Ser) showed a higher frequency of this variant in our control group (22.8%) than in the non-Finnish European group (12.9%; gnomAD v2.1.1. for rs7537203). We believe that this difference may be due to population differences. Indeed, in the non-Finnish European group only around 9% of the sample was composed by southern European individuals. In addition, there is no indication whether any Portuguese subjects were included in the sample. Data available in ensembl.org are similar. Information provided by this database indicates that the southern European group was only composed of Spanish individuals and Italian subjects from Tuscany (www.ensembl.org).

**Figure 8:**
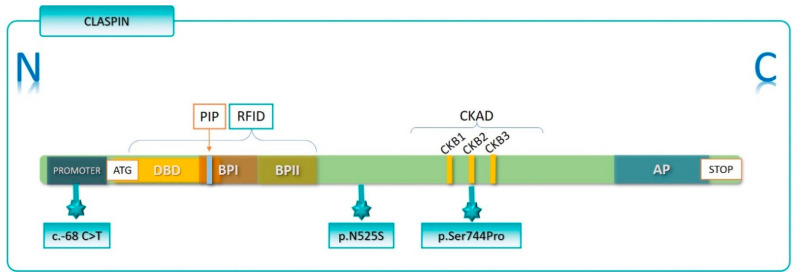
Claspin functional domains and location of the genetic variants. The N terminal end of Claspin contains the replication fork-interaction domain (RFID) (aa 273-622), which harbours two smaller regions rich in basic residues termed basic patches 1 (BP1) (aa 273–492) and 2 (BP2) (aa 492–622), and the PCNA-interacting domain (PIP). RFID mediates interactions with replication associated proteins and contributes to Claspin physical interaction with chromatin-containing stalled replication forks. The DNA binding domain (DBD) that spans aa 149 to aa 340 efficiently interacts with branched DNA. The C terminal end harbours an acidic patch (AP) that interacts with the DNA-binding domain (DBD) domain located in the N terminal region. The Chk1-activating domain (CKAD) is located in the C terminal spanning aa 837 to aa 1206. This region contains three 10 aa repeats phosphorylation sites in human Claspin (CKB1: aa 910-919; CKB2: aa 939-948 and CKB3: aa 976-985) that are CKγ1 kinase phosphorylation targets and are involved in Chk1 binding and activation.

As genetic variants have the potential to modify protein structure and/or function, we therefore used bioinformatics tools to predict the impact of the different genetic variants on Claspin function. *CLSPN* c.1574A>G, located on exon 8, causes an Asn to Ser substitution at position 525. Bioinformatics tools predicted p.Asn525Ser to be highly pathogenic and indicated that this variant could alter mRNA processing. Indeed, we observed that this variant increased exon 8 skipping in a pCAS2 minigene assay. We also showed that the p.Asn525Ser mutant is associated with reduced Chk1 S317 phosphorylation levels, and hence to decreased Chk1 activation (Figure 3a–c). This effect cannot be attributed to a higher Claspin instability, nor does it appear to be associated with p.Asn525Ser impact on modification of post-translational sites, since this variant does not fall in any phosphorylation, acetylation or ubiquitination residues. This variant is located in N-terminal domain of Claspin (Figure 8) that ranges from aa 265 to aa 605, and was identified in Xenopus as a replication fork-interaction domain (RFID) [24]. This region has been shown to be required for the interaction of Claspin with several replication-associated proteins, such as Cdc45, DNA polymerase ε, replication protein A and replication factor C complexes, and to be necessary for Claspin association with stalled replication forks.

**Figure 9:**
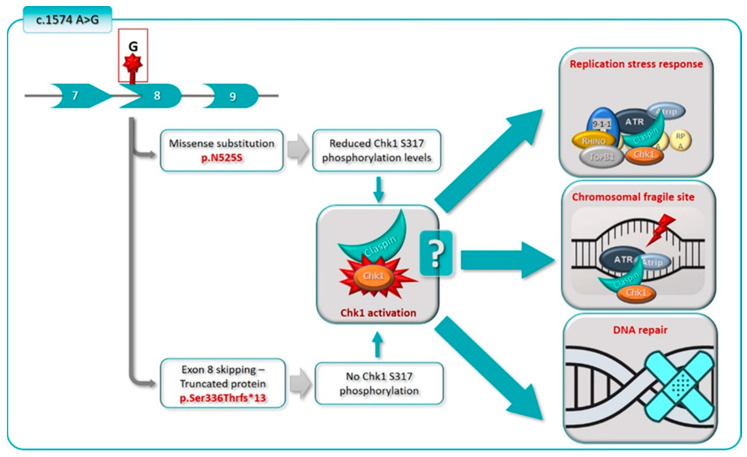
*CLSPN* c.1574A>G variants may impact multiple Claspin’s functions. p.Asn525Ser mutant is defective in its ability to phosphorylate Chk1, and can, therefore, affect checkpoint activation. In addition, as it is located at Claspin’s RFID, it may impact Claspin functions in DNA replication, such as maintenance of replication integrity, sensing of aberrant replication structures and the DNA replication process itself. This variant may also cause *CLSPN* exon 8 skipping, which leads to production of a 349 aa truncated Claspin protein (p.Ser336Thrfs*13) that completely lacks the C-terminal domain. Therefore, this isoform may lead to a defective Claspin’s response to DNA damage and replication checkpoint regulation.

In Xenopus, RFID contains two small regions rich in basic aa: the Basic Patch I (BPI), which ranges from aa 265 to aa 331, and BPII, from aa 470 to aa 600 [24]. RFID (aa 273–622), BPI (aa 273–492) and BPII (aa 492–622) are also found in human Claspin (Figure 8) [25]. BPI is involved in Claspin binding to chromatin but is not necessary for Claspin’s ability to mediate Chk1 activation. A DNA binding domain (DBD), ranging from aa 149 to aa 340, has been identified in human Claspin [21]. This region is highly conserved between several fungal and metazoan species. In Schizosaccharomyces pombe, it ranges from aa 160 to aa 317 [47], and has been shown to efficiently interact with branched DNA structures [21,47]. As for BPII, it has not only been implicated in the ability of Claspin to bind chromatin, but it also seems to be important for Chk1 phosphorylation [24]. Although Chk1 activation is mediated by a domain located at Claspin’s C-terminal designated Chk1-activating domain (CKAD) (aa 776 to 905, in Xenopus; and aa 837 to 1206 in humans), BPII appears to amplify Claspin-mediated Chk1 phosphorylation and, therefore, Claspin’s function in checkpoint regulation [24,25]. Thus, *CLSPN* p.Asn525Ser variant may interfere with Claspin’s ability to bind to chromatin, namely to stalled replication forks, and to mediate Chk1 phosphorylation during DNA damage or replication signalling. Furthermore, since BPII is involved in binding to Timeless and Cdc45, a co-factor for the replicative helicase, and has high affinity for fork-like DNA structures [24,25], *CLSPN* p.Asn525Ser variant can also impact Claspin function in DNA replication, such as the maintenance of replication integrity, sensing of aberrant replication structures, and the DNA replication process itself, although these processes were not assessed in this study (Figure 9).

*CLSPN* exon 8 skipping can generate a premature stop codon at position 336 (p.Ser336Thrfs*13) and lead to the production of a 349 aa truncated Claspin. To assess if such truncated protein could impact Claspin’s ability to mediate Chk1 activation, a Claspin truncated isoform (p.Ser336Thrfs*13) was produced. Claspin p.Ser336Thrfs*13 completely lacks the C-terminal domain and BPII, and part of the BPI, and is unable to mediate Chk1 phosphorylation (Figure 8). As already described, the C-terminal region of Claspin is primarily associated with the response to DNA damage and replication checkpoint regulation. This C-terminus contains the CKAD, which is both necessary and sufficient for Chk1 binding during checkpoint activation [24,25,48,49]. In human Claspin, this region consists of three repeats of 10 aa (aa 910–919; aa 939–948; and aa 976–985) [49], containing two particular phosphorylation sites, Thr916 and Ser945, presumably phosphorylated by CKγ1 kinase [50], that are required for Claspin-Chk1 binding and Chk1 phosphorylation during checkpoint activation [9,48].

More recently, Claspin C-terminal has been implicated in other functions, such as DNA replication. Claspin C-terminus contains a dense region of acidic aa, the Acidic Patch (AP) (aa 986–1100), which was implicated in DNA replication, namely, through regulation of origin firing and of Claspin binding to DNA [5]. Claspin has been shown to interact with Cdc7 via this AP domain and to be phosphorylated by Cdc7, this way promoting the phosphorylation and activation of the MCM protein complex by Cdc7 [26]. Interestingly, the C-terminus of Claspin also interacts with the DBD located at its N-terminal, masking a PCNA-interacting domain (PIP) and thereby suppressing Claspin’s DNA and PCNA binding ability. This intramolecular interaction is disrupted by Claspin phosphorylation by Cdc7, which allows the Claspin N-terminal region to bind to DNA and to replisome proteins, such as PCNA, Cdc45, Timeless [25,26], and its C-terminal to bind Cdc7, TopBP1, Pol δ, Rad-17-RFC and DNA polymerase ε [25,26]. Therefore, the absence of the C-terminus can affect not only the regulation of Chk1 functions, but also the role of Claspin in DNA replication (Figure 9).

We have also tried to evaluate the effects of exon 8 skipping in a more complex model such as HeLa cells, which harbour the c.1574A>G variant in heterozygosity. However, no evidence of altered splicing was detected for the transcripts obtained from these cells. This could be due to a small splicing effect that could not to be detected in vitro by the methodology used, or due to the elimination of the non-functional transcript by a protective physiological mechanism. In fact, as already mentioned, exon 8 skipping introduces a premature stop codon at position 336 (p.Ser336Thrfs*13). Transcripts containing premature termination codons (PTCs) can be recognised by a translation-dependent surveillance system, called Nonsense Mediated Decay (NMD), which involves activation of a series of enzymes, ultimately leading to the destruction of the abnormal transcripts [51,52]. It is therefore possible that we did not detect endogenous transcripts lacking exon 8 because they may have been degraded via the NMD pathway. In contrast to cellular transcripts, pCAS2 minigene-derived PTC transcripts are refractory to NMD given that this splicing reporter construct was deliberately designed to lack translation initiation codons [53]. This feature allows to specifically circumvent NMD, which facilitates the detection of aberrantly spliced transcripts, and may explain the different results obtained in the two systems. Nevertheless, another approach for future studies could be the inhibition of NMD, for instance by adding translation inhibitors (e.g., Puromycin or Cycloheximide) to the HeLa cell culture ~6 h before cell harvesting and RNA extraction.

The *CLSPN* promoter c.-68C>T variant has been detected in large-scale sequencing projects and can be assessed as rs372789882 in open genome aggregation databases, such as Ensembl, GnomAD, TopMed and 1000G, with reported frequencies, across populations, of less than 0.01 (MAF < 0.01). This variant has previously been detected in a study performed in our lab [39]. In the present study, c.-68C>T was detected in all groups (control, breast cancer, and glioma), but not in the cell lines analysed. No association with cancer has been found, and allelic and genotypic frequencies are similar across all groups, except for a slightly higher prevalence of the heterozygous genotype (CT) in glioma samples. Of note, we have not detected the mutant homozygous genotype (TT) in any of the groups screened. Nevertheless, no data regarding this variant’s functional impact, expression correlation or association with clinical features is currently available in the literature.

The pathogenicity of noncoding variants tends to be more difficult to assess than that of coding variants. However, promoter mutations have the potential to modulate gene transcription regulation and expression. Therefore, we decided to investigate whether the c.-68C>T variant could alter the activity of the *CLSPN* promoter. A 150 bp fragment of the *CLSPN* promoter surrounding the site of this genetic variation was inserted into a minimal promoter vector and luciferase assays were performed to assess the transcriptional activity associated with C and T alleles. We have observed a significantly higher transcriptional activity for the mutant T allele than for the WT C allele in both HEK293 and HeLa cells, but not in U2 OS cells (Figure 4). The differences observed between U2 OS cells and the other two cell lines used in this study can be possibly explained by a different molecular context, as expression of transcription factors may differ between cells of different tissues.

To investigate whether the observed increased transcriptional activity was associated with altered binding of transcription factors to *CLSPN*’s promoter, we performed EMSA. We found that the C and T alleles generate different band profiles, a finding that suggests differences in the composition of the transcription factor complex depending on the presence of the C or the T allele. Bioinformatics tools (TESS software, Philadelphia, PA, USA; TFSearch software, Connecticut, CT, USA and TFBind, Yokohama, Japan) were used to investigate whether this variant could alter the binding sequences of any transcription factor and yielded two candidate transcription factors: MyoD and myogenin. MyoD was selected as a candidate transcription factor due to a significant data linking MyoD to breast cancer [42,43,44]. We have decided to also investigate the E2F family of transcription factors due to previous work that reported that these proteins were involved in the regulation of *CLSPN* promoter activity [45,46]. However, our data suggest that none of these transcription factors is responsible for the distinct profiles detected by EMSA. This could mean that a yet unidentified transcription factor is responsible for the enhanced transcriptional activity observed. In fact, although database information (www.ensembl.org) and promoter analysis software predicted c.-68C>T to be located on a transcription factor binding site, no consensus was found regarding the transcription factor(s) implicated. It is also possible that the higher transcriptional activity of the T variant is due to elimination of a transcriptional repressive motif or with generation of an activator one.

Promoter mutations might be underrepresented in the set of variants underlying cancer predisposition due to difficulties in detection and in relating functional data with clinical features. For instance, deep sequencing of 360 primary breast cancers and advanced computational analysis of promoter regions, has identified nine genes harbouring recurrent mutations with potential to alter protein function [53]. In particular, FOXA1, an oncogene and driver of hormone receptor positive breast cancer, harbours a promoter mutational hotspot that causes FOXA1 overexpression via increased E2F binding. A model has been proposed in which a promoter mutation causes enhanced expression of FOXA1, this way promoting accessibility of oestrogen receptor binding sites, which allows cancer cells to grow under lower oestrogen conditions [54]. In addition, Fraile-Bettencourt and co-workers [55] have also identified genetic variants in the BRCA2 promoter that significantly up-regulated luciferase expression. These studies exemplify the importance of evaluating the impact of promoter mutations in cancer development. In line with these findings, we herein show that *CLSPN* c.-68C>T variant is associated with gain of function, although its cellular impact, namely with regard to net Claspin expression levels, was not assessed. Nevertheless, Claspin enhanced expression has been detected in a variety of cancer samples and cell lines and was associated with a worse prognosis [56,57,58,59]. Interestingly, Claspin overexpression, in coordination with that of Timeless, appears to constitute a cancer survival mechanism. Acting through a mechanism independent of its checkpoint function, when overexpressed in cancer cells, Claspin participates in a replication fork protection mechanism, which alleviates oncogene-driven replication stress and allows fork progression and, hence, cancer cell replication [35]. Of note, over-expression of proteins involved in DNA damage repair was also associated with metastatic progression in melanoma, as well as with therapeutic resistance [60,61]. It has been argued that metastatic cells require an efficient DNA damage response to acquire resistance to chemotherapy and to be able to invade and colonize other tissues [55].

Altogether, the herein described data along with current literature highlight the importance of Claspin in cancer and the need for further studying Claspin genetic variants and their biological impact.

## 4. Material and Methods

### 4.1. Patients and Samples

Peripheral blood samples from a total of 266 Portuguese Caucasian cancer patients were included in this study. More specifically, they were collected from 147 patients with a familial history of breast cancer (but negative for *BRCA1/2* mutations), 66 sporadic breast cancer patients and 53 glioma patients (average age of 57.2 ± 14.7 years, ranging from 22 to 78 years). The average age of the familial breast cancer group was 39.4 ± 11 years, ranging from 22 to 78 years, whereas sporadic breast cancer patients were all over 50 years old. A control group, consisting of healthy women with no oncologic history, was also included in the study (*n* = 79; average age of 65.9 ± 10 years, ranging from 51 to 94 years). All patients signed an informed consent for inclusion before they participated in the study. The study was conducted in accordance with the Declaration of Helsinki, and the protocol was approved by the Ethics Committee of the Portuguese Institute for Oncology at Coimbra (Instituto Português de Oncologia de Coimbra de Francisco Gentil, EPE; code of the project: *POCTI/MGI/48912/2002*). Patients characteristics are depicted in Table 4.

### 4.2. Cell Lines

U2 OS (ATCC^®^ HTB-96), HeLa (ATCC^®^ CCL-2), HEK293 (ATCC^®^ CRL-1573™) and HEK293T, RKO (ATCC^®^ CRL-2577), U-87MG (ATCC^®^ HTB-14) and DLD-1 (ATCC^®^ CCL-221) cell lines were derived from osteosarcoma, cervical cancer, human embryonic kidney cells, colon carcinoma, human primary glioblastoma and colorectal adenocarcinoma, respectively. All cell lines were acquired from ATCC. Cells were grown and maintained at 37 °C, 5% CO_2,_ in high glucose DMEM (Biowest, Nuaillé, France) supplemented with 10% heat-inactivated foetal bovine serum (PAA, Thermo Fisher Scientific, Waltham, MA, USA), 1% penicillin and streptomycin (Gibco, Thermo Fisher Scientific, Waltham, MA, USA) and 1 mM of Sodium Pyruvate (PAA, Thermo Fisher Scientific, Waltham, MA, USA).

### 4.3. Antibodies

The antibodies used were: anti-Chk1 (G-4), anti-Ku86 (C-20), and anti-MyoD (5.8A) from Santa Cruz Biotechnology, Dallas, TX, USA), anti-pSer317-Chk1 (Cell Signaling Technology, Boston, MA, USA), anti-HA (12CA) (Roche Applied Science, Mannheim, Germany), anti-Claspin NT [20], and anti-E2F (raised in E.W.-F.L. laboratory).

### 4.4. DNA Extraction

DNA was extracted using a standard phenol-chloroform procedure followed by ethanol precipitation. Briefly, cells were lysed in lysis buffer (10 mM Tris-HCl, (pH = 8.0); 0.1 M EDTA (pH = 8.0); 0.5% SDS; 20 μg/mL RNAse (Sigma-Aldrich, St. Louis, MO, USA); 0.5 μg/μL Proteinase K (Fermentas, Thermo Fisher Scientific)) and incubated at 56 °C, for 3 to 4 h, or overnight. After incubation, 1 volume of phenol/chloroform (1:1) was added to the solution and centrifuged at 3500 g for 10 min. This step was repeated as many times as required to obtain a clear interface. DNA was precipitated by adding 1/10 Sodium Acetate (3 M, pH = 5.2) and 2/3 volumes of ice-cold absolute ethanol. Precipitated DNA was washed in ethanol 70% and resuspended in ultra-pure water.

### 4.5. CLSPN Genotyping

The coding regions and exon-intron boundaries of *CLSPN* (reference sequence NM_022111) were screened for genetic alterations using intronic primers (available upon request). PCR reactions contained 200 ng of DNA, 1× Taq buffer, 200 μM of dNTPs, 10 μM of each primer and 2U of DreamTaq DNA Polymerase (Thermo Fisher Scientific) in a final volume of 50 μL. A standard thermocycler program was used to amplify all regions. Briefly, an initial denaturation step at 94 °C for 5 min was followed by 45 cycles of denaturation at 94 °C for 30 s, annealing at the specific primer temperature (60 °C for all primers, with the exception of 5’UTR + Exon 1 and Exon 10.2 primers for which the annealing temperature was 58 °C), for 30 s and an extension step at 72 °C for 40 s. A final extension step at 72 °C for 10 min was included. Electrophoresis in a 2% agarose gel stained with GelStar (Lonza, Basel, Switzerland) was used to check for amplification. The amplicons were purified using exonuclease I (Exo I) and shrimp alkaline phosphatase (SAP) (both from Fermentas, Thermo Fisher Scientific). Briefly, 1 μL of SAP and 0.5 μL of Exo I were added to approximately 50 μL of PCR product. The mixture was incubated for 30 min and enzymes were heat inactivated at 80 °C for 20 min. The purified products were then sent for sequencing (StabVida, Caparica, Portugal).

### 4.6. Bioinformatics Tools

To identify the *CLSPN* promoter we used the Promoter Prediction ProScan (University of Minnesota, Minneapolis, MN, USA), Softberry (Mount Kisco, NY, USA), MPromDb (University of Pennsylvania, PA, USA) and Promoter 2.0 Prediction Server (Lyngby, Denmark). TESS (Philadelphia, PA, USA), TFSearch (Connecticut, CT, USA) and TFBind (Yokohama, Japan) were used assess transcription factor binding to the *CLSPN* promoter. Prediction of the impact of nonsynonymous exonic *CLSPN* variants on protein structure and function was assessed using Polyphen-2. The QUEPASA method [40,41] and the Alamut Visual^®^ bioinformatics interface (Interactive Biosoftware, Rouen, France) were used to predict whether *CLSPN* variants could change splicing patterns. Phosphosite Plus was used to predict phosphorylation residues.

### 4.7. Luciferase Assay

To generate reporter construct for luciferase assays a genomic DNA sample heterozygous for c.-68C>T was used as a template for the PCR amplification of a 150 base pairs (bp) fragment of the *CLSPN* promoter. The forward and reverse primers contained tails corresponding to XhoI and HindIII restriction sequences, respectively, which allowed for the later digestion of the amplicon. After enzymatic digestion (Fermentas, Thermo Fisher Scientific), the amplicon was purified using Illustra GFX PCR DNA and gel band purification kit spin columns (GE Health Sciences, Chicago, IL, USA) and cloned into the Xho I and Hind III digested sites of the pGL4.23 minimal promoter vector (Promega, Madison, WI, USA). This plasmid contains the *LUC* reporter gene that codes for Luciferase, whose activity is measured in the luciferase assay. Cells were then transfected with the reporter constructs. The day before transfection, 0.5 × 10^5^ cells/well of HeLa and HEK293, and 1 × 10^5^ of U2 OS cells/well were plated in 24 well plates and incubated at 37 °C in a 5% CO_2_ atmosphere. Twenty-four hours later, 550 ng or 800 ng of wildtype (c.-68C+pGL4.23 vector), mutant (c.-68T+pGL4.23 vector) or empty (empty pGL4.23) vectors were transfected into HeLa cells or U2 OS cells, respectively. Two hundred nano-grams of β-galactosidase (β-Gal) vector were included in each transfection experiment for normalization purposes. Lipofectamine^®^2000 (Invitrogen, Thermo Fisher Scientific) was used to transfect all plasmids following the manufacturer’s instructions.

Twenty-four hours after transfection, cells were lysed with Passive Lysis Buffer (Promega) and luciferase activity was measured using the Luciferase Assay System kit (Promega) in a Tecan Infinite M200 luminescence reader. β-galactosidase activity was measured at 420 nm after a 30-min incubation with ONPG (Ortho-Nitrophenyl-β-Galactoside) (Sigma-Aldrich) at 37 °C. The assays were normalized by calculating the ratio between luciferase and β-gal activities. Three independent assays were performed in triplicates.

### 4.8. Fluorescent Electrophoretic Mobility Shift Assay (fEMSA)

To study the effect of the promoter variant on *CLSPN* promoter activity, we have used fEMSA. For that, we have first prepared nuclear extracts. The extraction of the nuclear fraction was performed as described [62]. Briefly, cells were scrapped in cold PBS and centrifuged at 1100 rpm for 5 min at 4 °C. Cells were then resuspended in membrane lysis buffer (10 mM HEPES (pH 8.0); 1.5 mM MgCl_2_; 10 mM KCl; 1 mM DTT; 1× Aprotinin/Leupeptin), and incubated, on ice, for 15 min. After addition of 1% NP-40, the solution was vigorously shaken and the nuclei pelleted, by centrifugation, at 13,000 × *g* for 3 min. The nuclear fraction was resuspended in extraction buffer (200 mM HEPES (pH 8.0); 1.5 mM MgCl_2_; 25% glycerol; 420 mM NaCl; 0.2 mM EDTA; 1 mM DTT; 1× Aprotinin/Leupeptin), and incubated, on ice, for 30 min with continuous shaking, followed by centrifugation at 13,000× *g* for 15 min. The nuclear extracts (supernatants) were quantified using the Pierce BCA Protein Assay Kit (Thermo Fisher Scientific) and either immediately used for fEMSA, or stored at −80 °C.

For fEMSA, non-labelled (cold probes) and 5′-Cy5 fluorescent-labelled oligonucleotides (Eurogentec, Seraing, Belgium) were mixed with their complementary oligonucleotides at a final concentration of 10 μM in annealing buffer (10 mM Tris, 1 mM EDTA, 50 mM NaCl (pH = 8.0)). The oligonucleotide mixtures were placed in a thermocycler, at 95 °C, for 10 min and cooled down to 22 °C at a rate of 1 °C per minute. The annealed duplexes were further diluted in water for a working solution of 6 μM. The oligonucleotide sequences used are shown in Table 5.

EMSA binding reactions were performed in a final volume of 20 μL containing 30 μg of HeLa nuclear extract and 5 μL of 5× EMSA binding buffer (100 mM Tris (pH 8.0); 250 mM KCl; 5 mM DTT; 5 mM EDTA; 50% Glycerol and 0.5 mg/mL acetylated BSA). For competition experiments, 0.2×, 0.5×, 1×, 2×, and 5× molar excess of cold competitor oligonucleotides were pre-incubated with nuclear extracts for 30 min at room temperature (RT). Next, 2 μL of Cy5-double labelled duplexes were added and incubated for further 30 min at RT. The samples were loaded without loading dye into a 1× TBE 4% non-denaturing polyacrylamide gel, run at 100 V, for 1 h, and scanned with the Molecular Imager FX (BioRad, Hercules, CA, USA). For supershift assays, 1 μL of antibodies against proteins of the E2F family, or 1 μL of anti-Myo-D antibody, were incubated with nuclear extracts for 30 min prior to the addition of the labelled oligonucleotide. Protein-DNA complexes were separated from free labelled probes by electrophoresis and read as previously described.

### 4.9. Minigene Splicing Assay

The minigene assay was performed according to the Gaildrat and co-workers [63] protocol. First, we prepared the *CLSPN* minigene constructs as follows. Wild type (WT) and mutant inserts were amplified by PCR from genomic DNA samples containing *CLSPN* c.1574A>G, c.2028+16G>A, c.2230T>C, c.3595-3597del and c.3839C>T variants. The c.1574A>G and c.3839C>T variants were studied in single and double exon contexts. The c.2230T>C variant was only analysed in double-exon context, while c.2028+16G>A and c.3595_3597del were only studied within single--exon constructs. The primers used (Table 6) were designed with tails carrying BamH I and Mlu I recognition sites. Primers were specifically designed to avoid SNPs and repetitive sequences, and to localize at approximately 150 bp from the exon. The obtained amplicons were digested, purified and introduced into the pCAS2 vector, previously digested with BamH I and Mlu I (Fermentas, Thermo Fisher Scientific). pCAS2 is an expression vector containing a SERPING1-derived splicing cassette with 2 exons (A and B) separated by an intron containing unique BamHI and MluI sites [53]. Expression of the cassette is driven by the CMV promoter. The minigene constructs were sequenced to assure that the variant of interest was present and that no other variants were introduced during the cloning process.

The minigene constructs were then transfected into HeLa cells using the FuGENE 6 transfection reagent (Roche Applied Science, Penzberg, Germany), according to the manufacturer’s instructions. Twenty-four hours after transfection, cells were collected and total RNA was extracted using the NucleoSpin RNA II kit (Macherey Nagel, Duren, Germany). 200 ng of RNA were used for Reverse Transcriptase PCR (RT-PCR) with OneStep RT-PCR kit (Qiagen, Venlo, The Netherlands) and the pCAS2 KO1F and pCAS2R primers (TGAGGTCGCCGCCCATCAC and ATTGGTTGTTGAGTTGGTTGTC, respectively) in a final reaction volume of 25 μL. The forward primer KO1F is specific for the pCAS2 vector sequence, allowing transcript discrimination. Splicing products originated from the WT and mutant constructs were resolved on a 2.5% agarose gel stained with ethidium bromide and visualized under UV light with saturating and non-saturating exposure. Finally, DNA bands were excised from the gel, purified and sequenced.

### 4.10. Transcripts Analysis by Reverse Transcriptase-PCR

*CLSPN* transcripts from HeLa cells, which contain heterozygous c.1574A>G, c.2028+16G>A and c.3839C>T variants were analysed by Reverse Transcriptase PCR (RT-PCR). HEK293 cells, which are WT at these positions, were used as control. RNA was extracted with TRIzol (Thermo Fisher Scientific), following the manufacturer’s instructions, and 200 ng of RNA were converted into cDNA using the SuperScript™ VILO™ cDNA Synthesis Kit protocol (Invitrogen, Thermo Fisher Scientific). The RT-PCR reaction consisted of 1 μL of cDNA, 1× Taq buffer, 200 μM of dNTPs, 2U of DreamTaq DNA Polymerase (Thermo Fisher Scientific) and 10 μM of each primer pair in a final volume of 50 μL. The sequences of the RT-PCR primers, which mapped to flanking exons, are listed on Table 7. The thermocycler program comprised an initial denaturation step at 94 °C for 5 min, followed by 35 cycles of amplification consisting of a denaturation step at 94 °C for 30 s; annealing at 64 °C for 30 s and an extension step at 72 °C for 50 s. A final extension step at 72 °C for 10 min was added. Amplification was checked by electrophoresis in a 1% agarose gel stained with GelStar (Lonza, Basel, Switzerland). RT-PCR products were purified using Exo I and SAP (both from Fermentas, Thermo Fisher Scientific), as described above, and sequenced (Stabvida, Caparica, Portugal).

### 4.11. Allelic Imbalance Analysis by Primer Extension Assay–SNaPshot

*CLSPN* c.1574A>G allele-specific expression was determined by SNaPshot assay. First, a semi-quantitative RT-PCR was performed using primers surrounding the relevant nucleotide. Briefly, 200 ng of RNA were reverse transcribed and amplified in a reaction mix containing 1× Qiagen buffer, 200 μM of dNTPs, 15 μM of each forward (5′-TAAACCCCGGCCCACTTGCC-3′) and reverse (5′-GGCTGATAGGATGGAATCGTGG-3′) primers, and 1 μL Qiagen One step RT-PCR enzyme mix in a final volume of 20 μL. The thermocycler program consisted on an initial step at 50 °C for 30 s and a denaturation step at 95 °C for 30 s, followed by 30 cycles of an amplification program consisting of 94 °C for 30 s, 68 °C for 30 s, 72 °C for 1 min and 20 s and a final extension step at 72 °C for 10 min. Electrophoresis in a 2% agarose gel stained with GelStar (Lonza, Basel, Switzerland) was used to check for amplification. Five microliters of the amplification product were purified with Exo I and SAP (both from GE Healthcare) and the reaction incubated for 1 h at 37 °C, followed by an enzyme inactivation step at 75 °C for 15 min.

The resulting product was used in an extension reaction performed with a primer designed to have a 3′ end located immediately upstream of the polymorphic nucleotide. The extension reaction occurred in a final volume of 10 μL containing 2.5 μL of SNaPshot^®^ Multiplex Ready Reaction Mix (Applied BioSystems, Foster City, CA, USA), 0.2 μM SNaPshot primer (5′ GTGATACTTGAACCTGAAACCA 3′), 1× BigDye Terminator buffer (Applied BioSystems) and 2 μL of purified PCR product. The reaction was submitted to 25 cycles of an extension program at 96 °C for 10 s, 50 °C for 5 s and 60 °C for 30 s. The reaction was purified as described before. Next, a denaturation step was performed. The denaturation reaction consisted of 2 μL of extension products, 8 μL of formamide and 0.5 μL of internal standard GeneScan-120LIZ size standard (Applied BioSystems). The reaction was denatured, at 95 °C for 2 min and 30 s, and immediately placed on ice. The reaction was loaded on a capillary sequencer (ABI PRISM Genetic Analyzers, Applied Biosystems) and analysed by PeakScanner (Applied BioSystems). Allelic imbalance measurement was achieved by measuring peak areas corresponding to the A and G alleles determined with the Peak Scanner software (Applied BioSystems). Peak areas correspond to the relative mRNA expression of each allele, this way allowing for the calculation of the mRNA ratio. The complementary DNA (cDNA) c.1574A/c.1574G ratio was normalized with the correspondent genomic DNA (gDNA) c.1574A/c.1574G ratio to correct for possible variations occurring from dye incorporation due to sequence composition. The same protocol was used to measure gDNA *CLSPN* c.1574A>G ratio. Briefly, HeLA gDNA was amplified in a PCR reaction containing 100 ng of template, 1× AccuPrime™ Pfx DNA Polymerase buffer, 15 μM of the forward (5′ TCACTAggatccCATGAACATTTAGTTTTGTAGC 3′) and reverse (5′ TGATAGacgcgtGCTTAGATCATTCTGATACC 3′) primers and 1.25U of AccuPrime™ Pfx DNA Polymerase (Life Technologies), in a final volume of 50 μL. The thermocycler was setup to perform two consecutive amplification programs: (i) an initial denaturation step, at 95 °C for 2 min, followed by 10 cycles of denaturation at 95 °C for 15 s; annealing at 60 °C, with a decrease of 0.5 °C, per cycle, for 20 s; and an extension step at 68 °C, for 1 min and 20 s; followed by (ii) 25 cycles of an initial denaturation step, at 95 °C, for 15 s; annealing, at 55 °C, for 20 s; an extension step, at 68 °C, for 1 min and 20 s; and a final extension step, at 68 °C, for 10 min.

### 4.12. Functional Assays

To study the functional impact of *CLSPN* c.1574A>G (p.N525S), we began by building the relevant constructs, as follows. The 2XHA Claspin construct previously described [64] was used to introduce silent mutations that are not recognized by the siRNA against Claspin (siRNA sequence with changes in lower case: GGAAcGgAAaGCAGCCAGA). The N525S and S950A (control) variant were introduced in the siRNA resistant plasmid using the QuickChange Site-Directed Mutagenesis Kit (Agilent Technologies, Santa Clara, CA, USA). Stable U2 OS clones expressing siRNA resistant WT or p.N525S variant were obtained after transfection, selection with G418 (Sigma-Aldrich), picking resistant colonies and checked for HA positive signal by Western blot. Both cell lines expressing stably either WT or p.N525S siRNA resistant versions of Claspin were transfected with siRNA against Claspin (sequence: GGAAAGAAAGGCAGCCAGAdTdT, purchased from Microsynth, Balgach, Switzerland) using lipofectamine RNAiMax (Thermo Fisher Scientific) following the manufacturer’s instructions. Forty-two hours after transfection cells were treated with UV light (40 J/m^2^) and collected at different times post-irradiation and extracts being analysed by Western blot.

*CLSPN* c.1574 truncated (p.Ser336Thrfs*13) construct was obtained by overlap PCR and TA cloning into a PCDNA3.1 plasmid. p.Ser336Thrfs*13 construction was based on two fragments (A and B). Fragment A, consisting of nucleotides 1 to 1004 (exon 1 to 7) of *CLSPN* isoform 1 mRNA (Nucleotide Sequence, 4020 nt: CCDS396); and Fragment B consisting of a duplex formed by two synthesized complementary oligonucleotides (Eurofins, Lisbon, Portugal) corresponding to the remaining sequence of p.Ser336Thrfs*13 and including 17 bp that matched fragment A in its 3′ extremity, for overlapping purposes. Fragment A was amplified by RT-PCR using cDNA from HeLa cells as the template, as described above (see Transcripts analysis by Reverse Transcriptase-PCR). Annealing was performed at 66 °C for 30 s. Fragment B consisted of two complementary oligonucleotides comprising 58 bp in length (Table 8) that were annealed in a 1:1 proportion to a final concentration of 10 μM in TE buffer. The mixture was heated to 99 °C for 5 min and cooled down to room temperature. Duplexes were visualized under UV light in a 2.5% agarose gel stained with GelStar (Lonza, Basel, Switzerland).

Fragments A and B were joined through an overlap PCR in a reaction mixture containing a 1:1 proportion of each fragment, 1× Taq buffer, 200 μM of dNTPs and 2U of DreamTaq DNA Polymerase (Thermo Fisher Scientific) in a final volume of 50 μL. No primers were added to this reaction. The thermocycling program consisted of an initial denaturation step at 94 °C for 2 min, followed by 15 cycles consisting of denaturation at 94 °C for 30 s; annealing at 68 °C for 30 s, an extension step at 72 °C for 1 min, and a final extension at 72 °C for 10 min. Next, 5 μL of the resulting PCR product was added to the PCR mixture containing the above reagents plus 15 μM of primer Fw1 and primer Rv2 (Table 8) and amplified using the previously described thermocycler program with 35 cycles. Amplification was checked by electrophoresis in a 1% agarose gel stained with GelStar (Lonza, Basel, Switzerland). PCR products of the expected size (1044 bp) were purified as previously described and sequenced. TA cloning into pCDNA3.1 plasmid and subcloning into a flag-plasmid were performed following the manufacturer’s instructions (RBC TA Vector System, Toronto, ON, Canada).

To study the functionality of the Ser336Thrfs*13 mutant, HEK293T cells were transfected with 5 μg of plasmids expressing Flag-Claspin WT or Flag-truncated Ser336 (p.Ser336Thrfs*13). All plasmids were transfected using the Ca-Phosphate method as described before [65] and twenty-four hours after transfection, cells were incubated with MG132 (5 μM) at 37 °C, overnight. The following day, cells were collected and lysed with a buffer containing 7 M Urea, 1% SDS, 150 mM NaCl and 50 mM Tris (pH = 8). Samples were then sonicated and proteins quantified using the BCA method. 40 μg of each extract were loaded into an 8% polyacrylamide gel and Western blots were carried out using anti-NT Claspin [64] and anti-Ku 86 (Santa Cruz Biotechnology, Dallas, TX, USA).

### 4.13. Statistical Analysis

Genotype and allele frequencies were compared using the χ^2^ test. Differences in exon skipping between alleles in the minigene assay and differences in luciferase activity between alleles were analysed using a One-way ANOVA with multiple a posteriori comparisons, using Bonferroni’s correction. The statistical analyses were performed using GraphPad Prism 5 software (San Diego, CA, USA). Differences were considered statistically significant if *p* < 0.05.

## 5. Conclusions

Claspin performs multiple functions central to cell homeostasis and genome protection. In this study, we have shown that Claspin variants may be associated with susceptibility to breast cancer and glioma development. Furthermore, we have demonstrated that some of these variants had a relevant functional impact. Identified *CLSPN* variants can alter Claspin availability and/or function by changing its expression levels (c.-68C>T promoter variant), altering mRNA processing (c.1574A>G exon 8 variant), and decreasing Chk1 phosphorylation and checkpoint activation. However, additional research is needed to clarify the impact of these variants, particularly in the context of cancer development.

## Figures and Tables

**Table 1:** *Claspin* gene (*CLSPN*) variants detected in breast cancer and glioma patients.

Variant	Rs Number	Gene Location	aa Change	fBC	sBC	Glioma	Controls
c.-68C>T	rs372789882	Promoter	n.a.	2/147	1/66	3/53	1/79
c.17G>A	rs61751002	Exon 1	p.Gly6Asp	16/147	7/66	6/53	8/79
c.1574A>G	rs7537203	Exon 8	p.Asn525Ser	36/147	10/66	14/53	28/79
c.2028+16G>A	rs535638	Intron 10	n.a.	74/147	56/66	52/53	68/79
c.2230T>C	rs753369867	Exon 12	p.Ser744Pro	2/147	1/66	1/53	0/79
c.3595-3597delGAA	rs200760879	Exon 22	p.Glu1199del	16/133	*	9/53	17/79
c.3839C>T	rs35490896	Exon 24	p.Ser1280Leu	19/133	*	9/53	19/79

aa, amino acid; n.a., not applicable, fBC, familial breast cancer; sBC, sporadic breast cancer; * These variants were not screened in sporadic breast cancer samples.

**Table 2:** Comparative analysis of germline *CLSPN* c.1574A>G and c.2028+16G>A variants between control individuals, and patients with breast cancer (familial and sporadic forms) or glioma tumours.

			c.1574A>G, p.Asn525Ser					c.2028+16G>A				
			Genotypic (*n*, %) and Allelic Frequencies (%)									
Group		*n*	AA	AG	GG	A	G	GG	GA	AA	G	A
**Control**		79	51 (64.6)	20 (25.3)	8 (10.1)	122 (77.2)	36 (22.8)	11 (13.9)	19 (24.1)	49 (62.0)	41 (25.9)	117 (74.1)
**Breast Cancer**	**Familial**	147	111 (75.5)	32 (21.8)	4 (2.7)	254 (86.4)	40 (13.6)	73 (49.6)	32 (21.8)	42 (28.6)	178 (60.5)	116 (39.5)
		*p* value	*p* < 0.05			*p* < 0.05		*p* < 0.0001			*p* < 0.0001	
	**Sporadic**	66	56 (84.9)	9 (13.6)	1 (1.5)	121 (91.7)	11 (8.3)	10 (15.1)	12 (18.2)	44 (66.7)	32 (24.2)	100 (75.8)
		*p* value	*p* < 0.05			*p* < 0.01		0.6916			0.7866	
**Glioma**		53	39 (73.6)	13 (24.5)	1 (1.9)	91 (85.8)	15 (14.2)	1 (1.9)	14 (26.4)	38 (71.7)	16 (15.1)	90 (84.9)
		*p* value	0.1699			0.1112		0.0615			*p* < 0.05	

**Table 3:** *CLSPN* variants detected in cell line samples.

*CLSPN* Variants							
Cell Line	c.-68C>T	c.17G>A	c.1574A>G	c.2028+16G>A	c.2230T>C	c.3595_3597del	c.3839C>T
HEK293	WT	WT	WT	AA	WT	WT	WT
HeLa	WT	WT	AG	GA	WT	WT	CT
RKO	WT	WT	WT	AA	WT	WT	WT
U2 OS	WT	AA	AG	GA	WT	WT	WT
U87-MG	WT	WT	WT	AA	WT	WT	WT
DLD-1	WT	WT	WT	AA	WT	WT	WT

WT, wild type.

**Table 4:** Characteristics of the patients included in the study.

Cancer Type	Number	Gender (Female/Male)	Age (Years, Mean ± SD)	Additional Information
Familial Breast Cancer	147	128/19	39.4 ± 11	Co-morbidities: bilateral (16), Ovary cancer (4), Medullary thyroid cancer (4), Colon cancer (3), Melanoma (1), Uterus/endometrial cancer (3), Larynx cancer (1), Gastric cancer (1), Prostate cancer (1)
Sporadic Breast Cancer	66	66/0	>50	
Glioma	53	24/29	57.2 ± 14.7	Grade: I (0); II (11); III (9); IV (33) Types: Oligo (9); Astro (38); Mix (4); Ependimoma (2)

Oligo: oligodendroglioma; Astro: Astrocytoma; Mix: tumour with mixed types.

**Table 5:** Oligonucleotide sequences used in fEMSA.

Oligonucleotide	Sense Strand Sequence
c.-68C>T **(C)**	ggagacggcgggagc**C**gctgctctccggctg
c.-68C>T **(T)**	ggagacggcgggagc**T**gctgctctccggctg

**Table 6:** Primers used to amplify the inserts used in the minigene assays constructs.

Variant	Exon (s)	Sequence	Amplicon (bp)
c.1574A>G	Exon 7 + exon 8	Fw–TCACTA**ggatcc**GCTTTTTTGTACTTAGCTCC Rv–TGATAG**acgcgt**GCTTAGATCATTCTGATACC	1200
c.1574A>G	Exon 8	Fw–TCACTA**ggatcc**CATGAACATTTAGTTTTGTAGC Rv–TGATAG**acgcgt**GCTTAGATCATTCTGATACC	911
c.2028+16G>A	Exon 10	Fw–TCACTA**ggatcc**CTGAGTAGTATACTATCTAGG Rv–TGATAG**acgcgt**TACAGATATTCAGTGGTACTG	652
c.2230T>C	Exon 11 + exon 12	Fw–TCACTA**ggatcc**GGGAAAATTATGTTGATAATGG Rv–TGATAG**acgcgt**AAACTGCAAAAAATAGACCAAG	687
c.3595_3597del	Exon 22	Fw–TCACTA**ggatcc**GTGTTTTGAGAAGGCTATACC Rv–TGATAG**acgcgt**GAACATAAAGTAAAACCAGCC	549
c.3839C>T	Exon 23 + exon 24	Fw–TCACTA**ggatcc**GTGTCTCTTCTTGGAGCC Rv–TGATAG**acgcgt**GAAAGATAAACTTTCTCGGC	871
c.3839C>T	Exon 24	Fw–TCACTA**ggatcc**TTAATGTCAAAGGAGTCTGC Rv–TGATAG**acgcgt**GAAAGATAAACTTTCTCGGC	503

Sequences in bold correspond to the restriction sequences of BamH I and Mlu I.

**Table 7:** Primers used for RT-PCR amplification of *CLSPN* transcripts containing c.1574A>G, c.2028+16 G>A and c.3839C>T variants.

Variant	Primers Sequence	Primer Location	Amplicon (bp)
c.1574 A>G (exon 8)	Fw-TAAACCCCGGCCCACTTGCC	Exon 2	822
	Rv–AGCTTTTCACCTGGTTTTGTGTGGC	Exon 9/10	
c.2028+16 G>A (intron 10)	Fw-GCCACACAAAACCAGGTGAAAAGCT	Exon 9/10	607
	Rv-GGCTGATAGGATGGAATCGTGG	Exon 13	
c.3839 C>T	Fw-GATGAGGCAGAGGTGTCAGG	Exon 19	855
	Rv-TTAGCTCTCCAAATATTTGAAGATGC	Exon 25	

**Table 8:** Primers used to amplify the fragments used for overlap PCR.

Fragment	Primer Sequence (5′ to 3′)		Size (bp)
A	Fw 1	ATGACAGGCGAGGTGGGTTC	1004
	Rv 1	TTCAATAGTGCCATGGCATTTCC	
B	Oligo 1	GCCATGGCACTATTGAAAACTGGAAGCCTTGAAGCAGCGTTTCTGGAAGCATGCTAA	59
	Oligo 2	CGGTACCGTGATAACTTTTGACCTTCGGAACTTCGTCGCAAAGACCTTCGTACGATTT	
Overlap PCR	Fw 1	ATGACAGGCGAGGTGGGTTC	1044
	Rv 2	TTAGCATGCTTCCAGAAACGCTG	

## Supplementary Materials

The following are available online at https://www.mdpi.com/2072-6694/12/9/2396/s1, Figure S1: Detailed information about western blot in Figure 1, Figure S2: Detailed information about western blot in Figure 3, Figure S3: Detailed information in Figure 5, Figure S4: Competition assays indicate that *CLSPN* c.-68 C>T variant might alter transcription factor binding stoichiometry, Figure S5: Detailed information in Figure S4, Figure S6: Detailed information in Figure 6, Figure S7: Detailed information in Figure 7, Table S1: Comparative analysis of germline *CLSPN* c.-68C>T, c.17G>A and c.2230T>C variants in control, breast cancer (familial and sporadic forms) and glioma samples, Table S2: Comparative analysis of germline *CLSPN* c.3595-3597del and c.3839C>T variants between control, familial breast cancer and glioma samples.
