# Supplementary material for: Implications of CLSPN Variants in Cellular Function and Susceptibility to Cancer
Source: Cancers (Basel). 2020 Aug 24;12(9):2396. doi: 10.3390/cancers12092396 (PMC7565888; doi:10.3390/cancers12092396)
Supplement: Supplementary file 1 [file cancers-12-02396-s001.pdf]

Supplementary Materials

Implications of *CLSPN* Variants in Cellular Function and Susceptibility to Cancer

Diana Azenha, Santiago Hernandez-Perez, Yuse Martin, Marta S. Viegas, Alexandra Martins, Maria C. Lopes, Eric W.-F. Lam, Raimundo Freire and Teresa C. Martins

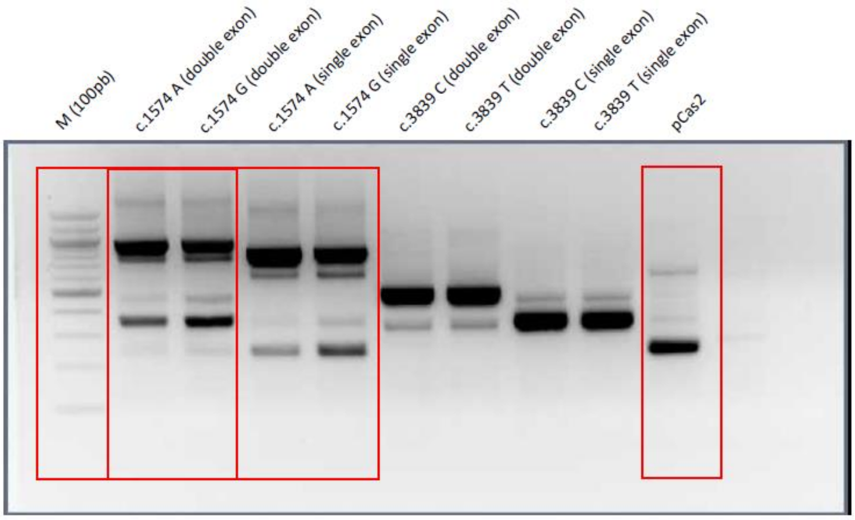

Figure S1. Detailed information about western blot in Figure 1.

Figure 3A

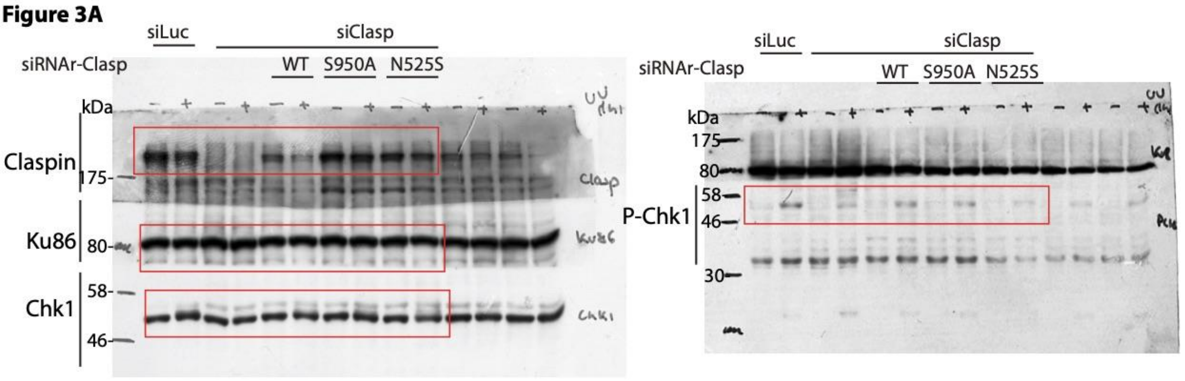

Figure 3B

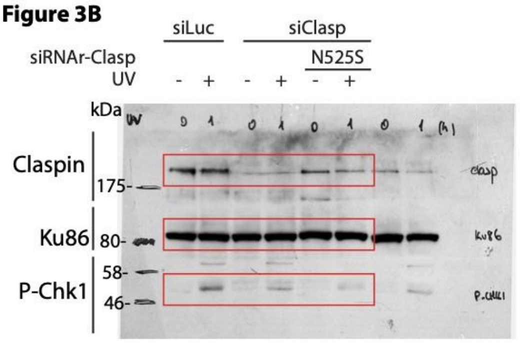

Figure 3C

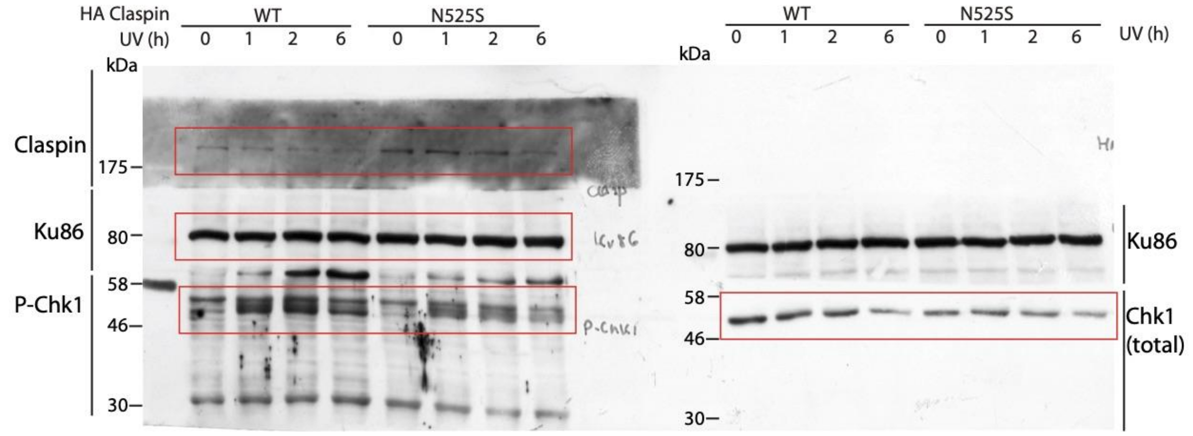

Figure 3D

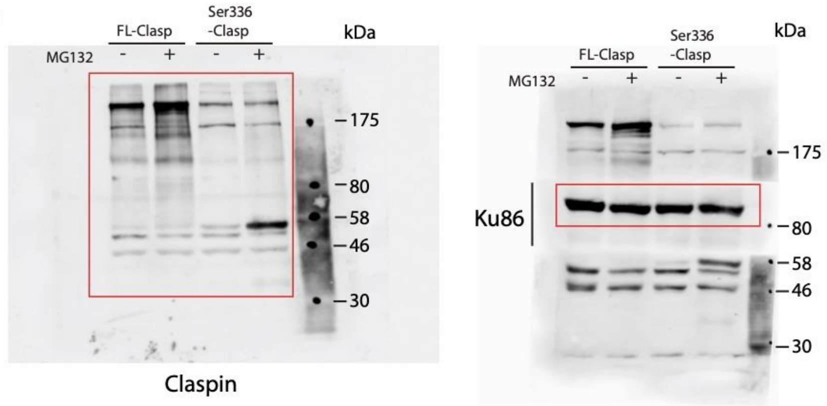

Figure S2. Detailed information about western blot in Figure 3.

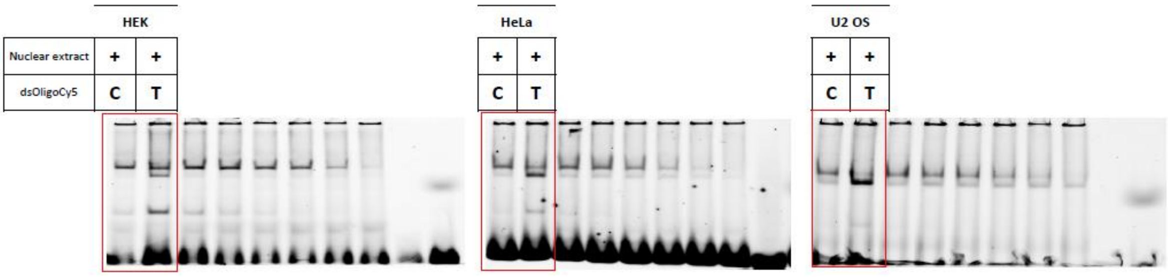

Figure S3. Detailed information in Figure 5.

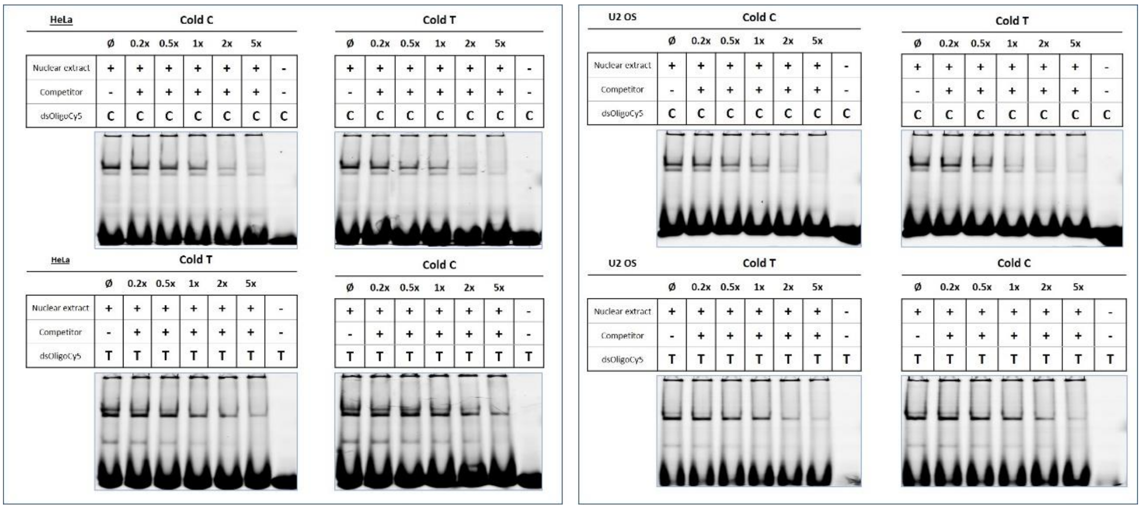

Figure S4. Competition assays indicate that CLSPN c.-68 C>T variant might alter transcription factor binding stoichiometry. Competition assays performed with HeLa and U2 OS nuclear extracts using labeled oligonucleotides (dsOligoCy5) containing C or T allele corresponding to CLSPN c.-68 C>T site. Where indicated (+ sign) cold Competitor probes containing either C or T were added to the reaction in a 0.2-, 0.5-, 1-, 2- or 5-fold excess and its effect compared to the null reaction (Ø lane—no competitor added). The last lane of each assay corresponds to a reaction in which only labeled oligonucleotide was added (ctrl–labeled oligonucleotide only). The assays were repeated at least three times for each cell line.

HeLa

Cold C

	Ø	0.2x	0.5x	1x	2x	5x	
Nuclear extract	+	+	+	+	+	+	-
Competitor	-	+	+	+	+	+	-
dsOligoCy5	C	C	C	C	C	C	C

Cold T

Ø	0.2x	0.5x	1x	2x	5x	
+	+	+	+	+	+	-
-	+	+	+	+	+	-
C	C	C	C	C	C	C

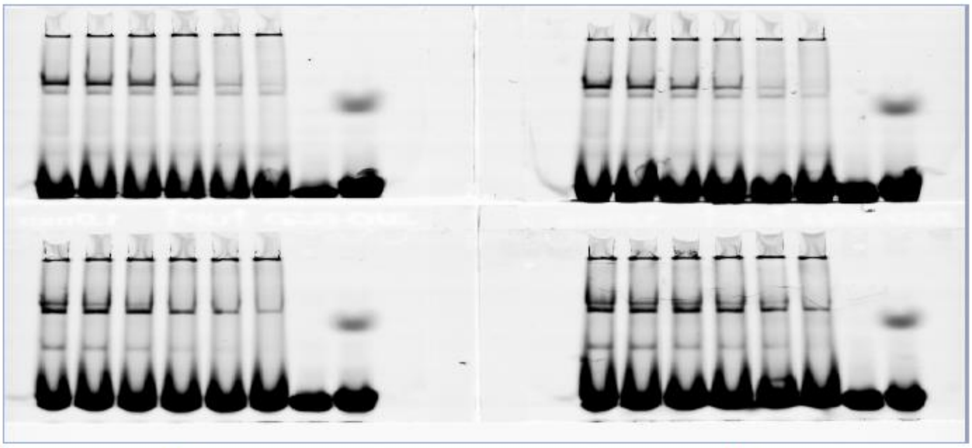

HeLa

Cold T

	Ø	0.2x	0.5x	1x	2x	5x	
Nuclear extract	+	+	+	+	+	+	-
Competitor	-	+	+	+	+	+	-
dsOligoCy5	T	T	T	T	T	T	T

Cold C

Ø	0.2x	0.5x	1x	2x	5x	
+	+	+	+	+	+	-
-	+	+	+	+	+	-
T	T	T	T	T	T	T

U2 OS

Cold C

	Ø	0.2x	0.5x	1x	2x	5x	
Nuclear extract	+	+	+	+	+	+	-
Competitor	-	+	+	+	+	+	-
dsOligoCy5	C	C	C	C	C	C	C

Cold T

Ø	0.2x	0.5x	1x	2x	5x	
+	+	+	+	+	+	-
-	+	+	+	+	+	-
C	C	C	C	C	C	C

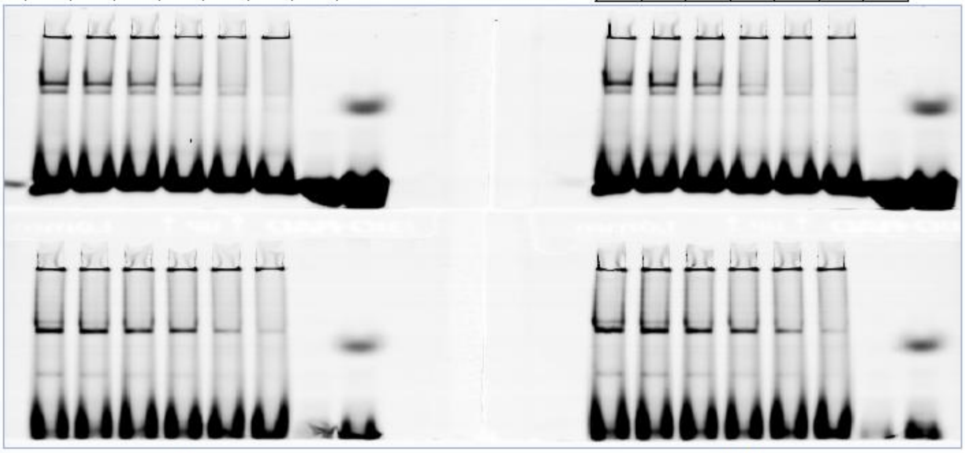

U2 OS

Cold T

	Ø	0.2x	0.5x	1x	2x	5x	
Nuclear extract	+	+	+	+	+	+	-
Competitor	-	+	+	+	+	+	-
dsOligoCy5	T	T	T	T	T	T	T

Cold C

Ø	0.2x	0.5x	1x	2x	5x	
+	+	+	+	+	+	-
-	+	+	+	+	+	-
T	T	T	T	T	T	T

Figure S5. Detailed information in Figure S4.

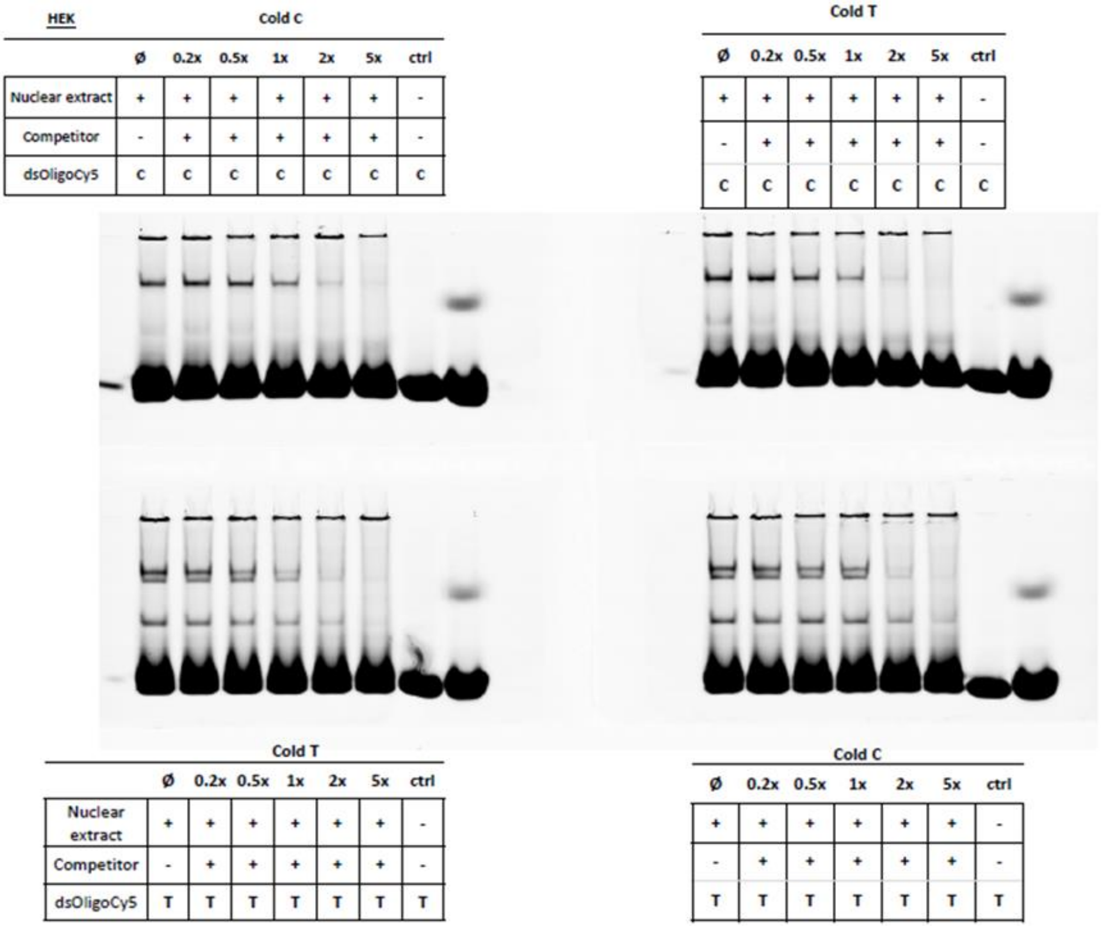

Figure S6. Detailed information in Figure 6.

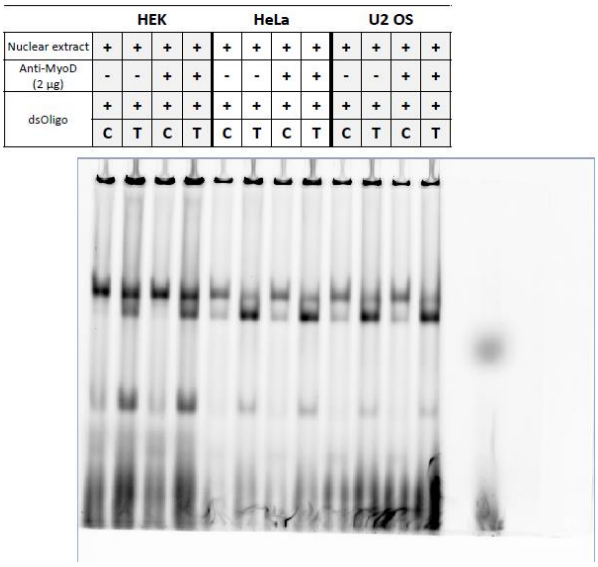

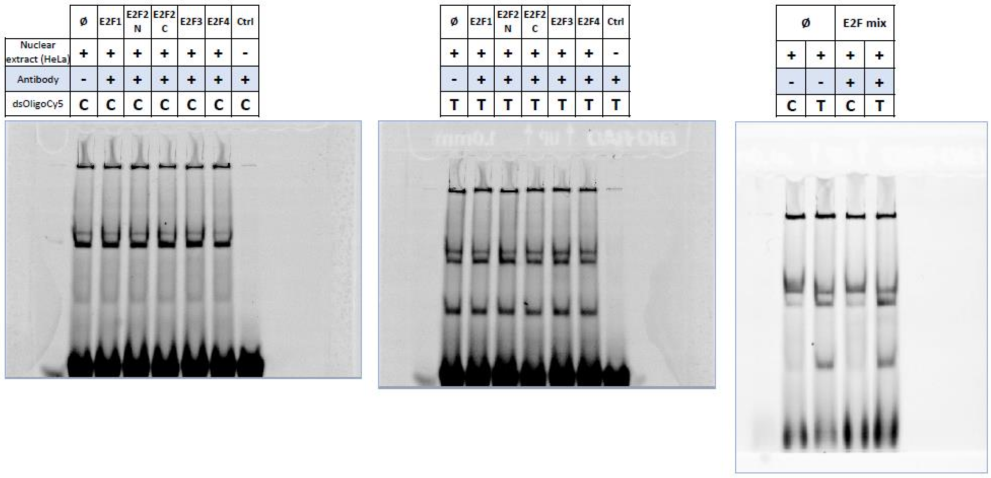

Figure S7. Detailed information in Figure 7.

Table S1. Comparative analysis of germline *CLSPN* c.-68C>T, c.17G>A and c.2230T>C variants in control, breast cancer (familial and sporadic forms) and glioma samples.

			**Genotypic (*n*, %) and Allelic Frequencies (*n*, %)**														
			**c.-68C>T**					**c.17G>A, p.Gly6Asp**					c.2230T>C. p.Ser744Pro				
**Group**		**Sample Number**	**CC**	**CT**	**TT**	**C**	**T**	**GG**	**GA**	**AA**	**G**	**A**	**TT**	**TC**	**CC**	**T**	**C**
**Normal**		79	78 (97.4)	1 (2.6)	0	157 (99.4)	1 (0.6)	71 (89.9)	7 (8.9)	1 (1.2)	149 (94.3)	9 (5.7)	79 (100)	0	0	158 (100)	0
**Breast**	**Familial**	147	145 (98.6)	2 (1.4)	0	292 (98.6)	2 (1.4)	131 (89.1)	16 (10.9)	0	278 (94.6)	16 (5.4)	145 (98.6)	2 (1.4)	0	292 (99.3)	2 (0.7)
		p value	0.8248			1.0		1.0			1.0		0.6428			0.5443	
	**Sporadic**	66	65 (98.5)	1 (1.5)	0	131 (99.2)	1 (0.8)	59 (89.4)	7 (10.6)	0	125 (94.7)	7 (5.3)	65 (98.5)	1 (1.5)	0	131 (99.2)	1 (0.8)
		p value	1.0			1.0		0.8248			1.0		1.0			0.4552	
**Glioma**		53	50 (94.3)	3 (5.7)	0	103 (97.2)	3 (2.8)	47 (88.7)	6 (11.3)	0	100 (94.3)	6 (5.7)	52 (98.1)	1 (1.9)	0	105 (99.1)	1 (0.9)
		p value	0.8248			0.3055		0.7			1.0		1.0			0.4015	

Table S2. Comparative analysis of germline *CLSPN* c.3595-3597del and c.3839C>T variants between control, familial breast cancer and glioma samples*.

		**Genotypic (*n*, %) and Allelic Frequencies (%)**									
		**c.3595-3597del, p.DelGlu1199**					**c.3839 C>T, p.Ser1280Leu**				
**Group**	***n***	**Wt**	**Het**	**Hom**	**wt**	**Del**	**CC**	**CT**	**TT**	**C**	**T**
**Normal**	79	62 (78.5)	16 (20.3)	1 (1.3)	140 (88.6)	18 (11.4)	60 (75.9)	18 (22.8)	1 (1.3)	138 (87.3)	10 (12.7)
**Familial Breast Cancer**	133	117 (88.0)	14 (10.5)	2 (1.5)	248 (93.2)	18 (6.8)	114 (85.7)	17 (12.8)	2 (1.5)	245 (92.1)	21 (7.9)
	p value	0.1450			0.1072		0.1654			0.8458	
**Glioma**	53	44 (83.0)	9 (17)	0	97 (91.5)	9 (8.5)	44 (83)	9 (17)	0	97 (91.5)	9 (8.5)
	p value	0.6277			0.5366		0.7			0.6347	

*These variants were not screened in sporadic breast cancer samples. Wt, wild type; Het, heterozygous; Hom, homozygous; Del, deletion.

© 2020 by the authors. Licensee MDPI, Basel, Switzerland. This article is an open access article distributed under the terms and conditions of the Creative Commons Attribution (CC BY) license (http://creativecommons.org/licenses/by/4.0/).
